# Increased Mortality Risk in Autoimmune Hepatitis: A Nationwide Population-Based Cohort Study With Histopathology

**DOI:** 10.1016/j.cgh.2020.10.006

**Published:** 2020-10-14

**Authors:** Rajani Sharma, Elizabeth C. Verna, Jonas Söderling, Bjorn Roelstraete, Hannes Hagström, Jonas F. Ludvigsson

**Affiliations:** * Center for Liver Disease and Transplantation, Division of Digestive and Liver Diseases, Columbia University Irving Medical Center, New York, New York; ‡ Division of Digestive and Liver Diseases, Department of Medicine, Columbia University College of Physicians and Surgeons, New York, New York; § Unit of Clinical Epidemiology, Department of Medicine, Solna, Karolinska Institutet, Stockholm, Sweden; || Unit of Hepatology, Center for Digestive Diseases, Karolinska University Hospital, Stockholm, Sweden; ¶ Department of Gastroenterology, Faculty of Medicine and Health, Örebro University, Örebro, Sweden; # Department of Pediatrics, Örebro University Hospital, Örebro, Sweden; ** Division of Epidemiology and Public Health, School of Medicine, University of Nottingham, United Kingdom

**Keywords:** Histopathology, Epidemiology, Death, Autoimmune Liver Disease

## Abstract

**BACKGROUND AND AIMS:**

Autoimmune hepatitis (AIH) is a chronic inflammatory liver disease that may lead to cirrhosis and liver failure, but data on overall mortality in AIH are conflicting.

**METHODS:**

This was a nationwide population-based cohort study in Sweden from 1969–2017 of 6,016 adults with AIH and 28,146 matched general population reference individuals. AIH was defined by a combination of a medical diagnosis of AIH plus a liver biopsy from any of Sweden’s 28 pathology departments. Through Cox regression, we estimated hazard ratios (HRs) for overall and cause-specific death. Liver transplant was included in our main outcome of death.

**RESULTS:**

During follow-up, 3,185 individuals with AIH died (41.4/1000 person-years) compared with 10,477 reference individuals (21.9/1000 person-years). The 10-year cumulative incidence of death was 32.3% (95%CI = 31.1–33.6) for AIH individuals and 14.1% (95%CI = 13.7–14.5) for reference individuals. This corresponded to an adjusted HR of 2.29 (95%CI = 2.17–2.41), which remained elevated ≥20 years follow-up. AIH individuals with cirrhosis on biopsy had a high risk of death (HR = 4.55; 95%CI = 3.95–5.25), while mortality in patients with fibrosis, inflammation without fibrosis, or necrosis did not differ. Portal hypertension and overlap with cholestatic liver diseases were also associated with death. AIH was associated with an increased risk of death from cardiovascular disease (HR = 1.27; 95%CI = 1.15–1.40), liver disease (HR = 66.24; 95%CI = 48.19–91.03) and extrahepatic malignancy (HR = 1.69; 95%CI = 1.51–1.89). In a sibling comparison, AIH individuals remained at increased risk of death.

**CONCLUSION:**

AIH is associated with a 2-fold increased risk of death. Risks were particularly high in individuals with cirrhosis, portal hypertension, and overlap with cholestatic liver disease.

Autoimmune hepatitis (AIH) is a chronic inflammatory liver disease that can progress to cirrhosis and liver failure,^1^ but earlier studies as to whether this translates into an increased risk of death are contradictory, such as increased mortality^2–6^ or no increase.^7–9^ Data also differ on whether cirrhosis influences mortality in AIH.^2,3,5,7–9^ Most prior studies have been limited in size, single center, and not population based.

Through the ESPRESSO (Epidemiology Strengthened by histoPathology Reports in Sweden) cohort, we have linked nationwide histopathology data with clinical data in the Swedish National Healthcare Registers.^10^ Given that the diagnosis of AIH requires liver biopsy,^1,11^ the ESPRESSO cohort offers a unique opportunity to enhance our identification of AIH individuals with the presence of liver biopsy in addition to clinical diagnosis codes, and provides the largest population-based sample to date with long-term follow-up. In this cohort study, we aimed to determine the risk of death in individuals with AIH compared with the general population and siblings, and perform key subanalyses by fibrosis stage.

## Materials and Methods

### ESPRESSO Cohort

All 28 pathology departments in Sweden were contacted to obtain histopathology report data from the gastrointestinal tract, including the liver.^10^ Through the unique personal identity number assigned to all Swedish residents,^12^ study participants were linked to Swedish Health Registers.

### Individuals With AIH

**Figure 1.**
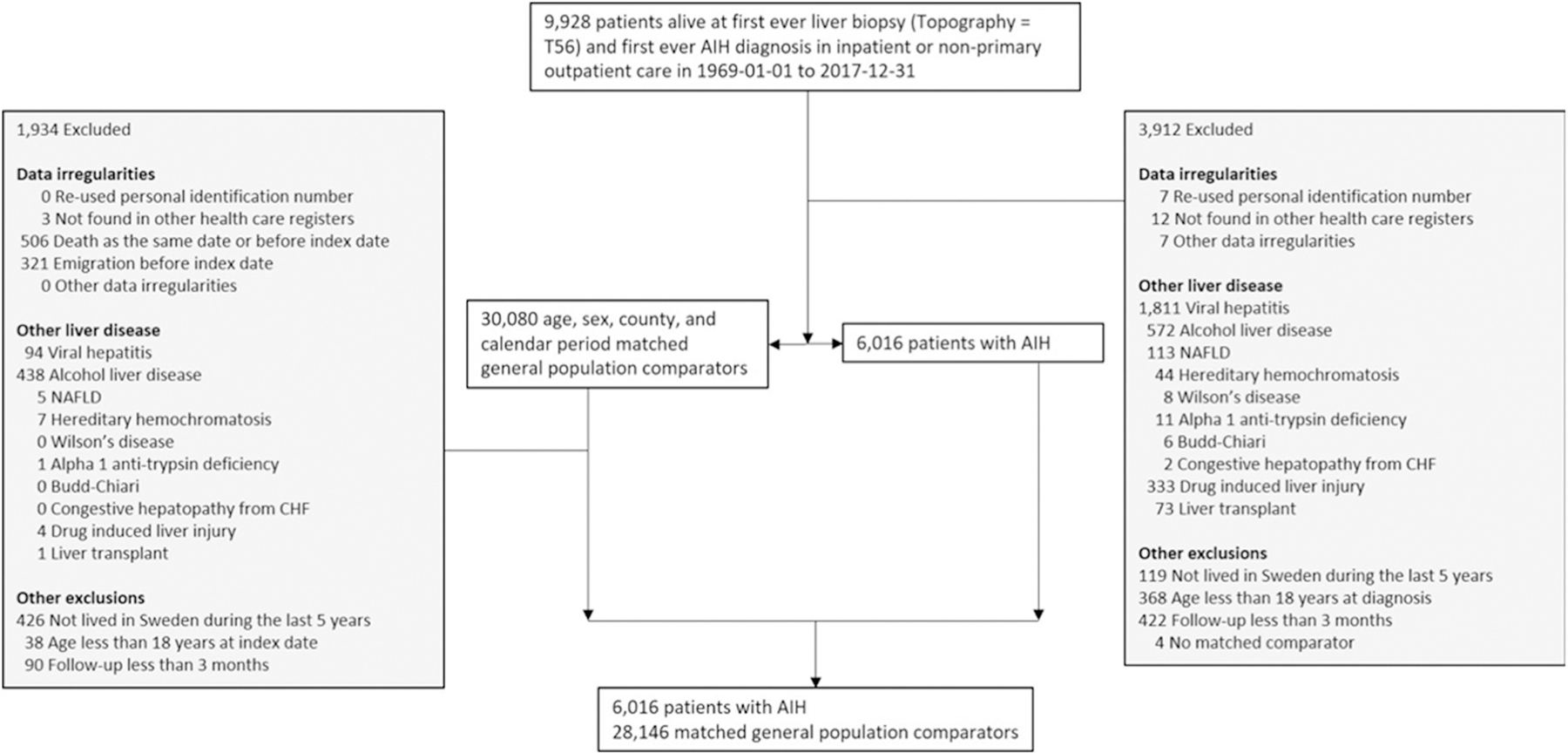
Flowchart of individuals with AIH and their matched general population comparators. CHF, congestive heart failure; NAFLD, nonalcoholic fatty liver disease.

We included individuals ≥18 years of age with a diagnosis of AIH determined both by the presence of an International Classification of Diseases (ICD) code for AIH and liver histopathology report data between January 1, 1969, and December 31, 2017 (Figure 1, Supplementary Table 1). By requiring liver biopsy data, we strengthened our ability to identify AIH individuals given that liver biopsy is required for diagnosis.^1,11^ Both inpatient and hospital-based outpatient diagnoses (available starting 2001) were included. Only patients who were alive at the time of liver biopsy were included to eliminate autopsy biopsies.

To further enhance the identification of AIH individuals, individuals with ICD codes for other chronic liver diseases at or before start of follow-up were excluded (Supplementary Table 1, Figure 1). Individuals were also excluded if they had a liver transplant before or at the time of liver biopsy, had not lived in Sweden over the past 5 years or formally emigrated, had follow-up <3 months after AIH diagnosis, had no matched comparator, or had other data irregularities (Figure 1).

Liver biopsy could occur either before or after ICD code diagnosis. Time of diagnosis of AIH was determined as the second of either liver biopsy or AIH ICD code date to avoid immortal time bias.

To assess the accuracy of our criteria for AIH, we performed a review of 100 random histopathology free-text reports of individuals who met our criteria from the full study period. Of these 100, 96 had sufficient information to be reviewed. A total of 92 of 96 had a histopathology report consistent with AIH resulting in a 96% positive predictive value (PPV) (95% confidence interval CI, 89%–99%). We also determined that 82 of 100 random individuals in our cohort (82%; 95% CI, 73%–88%) had at least 2 ICD codes for AIH.

### Population Reference Individuals and Siblings

For each individual with AIH, there were 5 matched reference individuals from the general population ≥18 years of age, matched on age, sex, county, and calendar year. Reference individuals were subject to the same exclusion criteria as individuals with AIH (Figure 1). In a separate sensitivity analysis, all full siblings were used as comparators and were also subject to the same exclusion criteria.

### Histopathology Report Data

Histopathology report data were available for each individual in the ESPRESSO cohort. As mutually exclusive categories, stage F4 fibrosis or cirrhosis, stage F1–F3 fibrosis, inflammation but without fibrosis, and other including normal biopsy were defined using SNOMED (Systemized Nomenclature of Medicine) codes based on the Batts-Ludwig criteria (Supplementary Table 2). Liver necrosis was not mutually exclusive (Supplementary Table 2). In Sweden, biopsy specimens are classified according to the SNOMED system developed by the National Health Service (United Kingdom) and the College of American Pathologists, allowing for standardization of liver biopsy data across all pathology departments in Sweden.^10^

### Mortality Outcome

The primary outcome was death or incident liver transplantation hereby called “death” (and being the inverse of “transplant-free survival”). Given that liver transplantation changes the natural history of chronic liver disease by improving survival, liver transplant was included in the primary outcome.^13^ Mortality data were obtained from the Total Population Register, and liver transplant data from the Patient Register (Supplementary Table 3). Secondary outcomes were cause-specific mortality obtained from the Cause of Death Registry (Supplementary Table 3).^14^

### Statistical Analyses

#### Main Analyses.

Kaplan-Meier failure curves with death as the failure event were determined for all-cause mortality (including liver transplantation) and cause-specific mortality (liver-related death included liver transplantation) between individuals with AIH and reference individuals. The incidence rates of all-cause and cause-specific mortality were reported with a 95% CI.

In each set of main analyses and subanalyses, we first performed Cox regression conditioned on age, sex, county, and calendar year of biopsy to estimate hazard ratios (HRs) for death in individuals with AIH compared with the matched general population reference individuals. We then added education and the following baseline medical comorbidities to our model: cardiovascular disease (CVD), extrahepatic cancer, diabetes, end-stage renal disease, other autoimmune diseases, and conditions that affect the use of first-line treatment (Supplementary Table 4).

In all main analyses, follow-up started at 3 months after AIH diagnosis date. We excluded 0–<3 months’ follow-up to reduce misclassification and rule out that liver biopsies were obtained from autopsies or terminally ill patients. Individuals were censored if they died, underwent liver transplantation, emigrated, or reached the last follow-up time of December 31, 2017. Reference individuals and sibling comparators were additionally censored if they developed a diagnosis of AIH.

Statistical significance was defined as 95% CI for risk estimates that did not include 1.0. SAS version 9.4 (SAS Institute, Cary, NC) statistical software was used to perform all analyses.

#### Subanalyses.

In prespecified subanalyses, we stratified by age at diagnosis, follow-up time, sex, year of diagnosis, country of birth, education, histopathology, portal hypertension, acute or subacute liver failure, overlap syndromes with primary sclerosing cholangitis (PSC) or primary biliary cholangitis (PBC), other autoimmune diseases, presence of conditions that affect use of first-line medications, and comorbidities (Supplementary Table 4). We also performed analyses within AIH individuals stratified by presence of necrosis, PBC or PSC overlap, portal hypertension, or the use of second-line medications.

Medications were determined from the Swedish Prescribed Drug Register, which began in July 2005.^15^ Proportions of individuals with an AIH diagnosis from July 1, 2006, onward with the first ever prescription of prednisone or prednisolone, azathioprine, and second-line medications (mycophenolate mofetil, 6-mercaptopurine, tacrolimus, or cyclosporine) were determined.^1^ In Cox regression analyses, medications were defined as time-varying covariates using methods described in Jarrick et al.^16^

#### Sensitivity Analyses.

We carried out a number of sensitivity analyses: first, using only mortality as the primary outcome (not including liver transplantation), and second, including individuals with 0–<3 months’ follow-up. Third, to reduce intrafamilial confounding, we examined mortality in AIH patients and their sibling comparators. Fourth, we restricted analyses to individuals diagnosed 1997–2017 (ie, during the ICD–Tenth Revision [ICD-10] era given more specific codes for AIH and viral hepatitis). Fifth, to further confirm that the liver biopsy was performed as part of the AIH workup, we restricted analyses to individuals with liver biopsy and AIH ICD code within 1 year. Sixth, to avoid a disproportionate contribution of shorter follow-up in later years of diagnosis and older age groups, we restricted the Cox regression analyses to 5-year follow-up in our calendar period and age group–specific analyses. Seventh, owing to concerns that some AIH individuals may have undergone liver biopsy as part of workup for cancer, we reran our analyses excluding individuals with an ICD code for any cancer before or at the time of diagnosis.

### Ethics

The ESPRESSO cohort was reviewed by the Stockholm Ethics Board (No. 2014/1287-31/4) approved on August 27, 2014.

## Results

We identified 9928 individuals with AIH. Of these, 3912 individuals were excluded. The final cohort consisted of 6016 individuals with AIH and 28,146 general population reference individuals matched on age, sex, county, and calendar year of biopsy (Figure 1). The mean age of individuals with AIH was 53.5 (SD = 16.3) years, and 60.6% were female. The median follow-up time was 9.5 (interquartile range, 4.2–21.0) years, and 48.1% had a follow-up time ≥ 10 years (Table 1). On liver biopsy, 34.4% had inflammation without fibrosis, 21.2% had fibrosis, 13.7% had cirrhosis, and 2.5% had necrosis; 4.9% had portal hypertension and 2.6% had liver failure.

### Mortality Compared With Reference Individuals

#### Overall Mortality.

**Figure 2.**
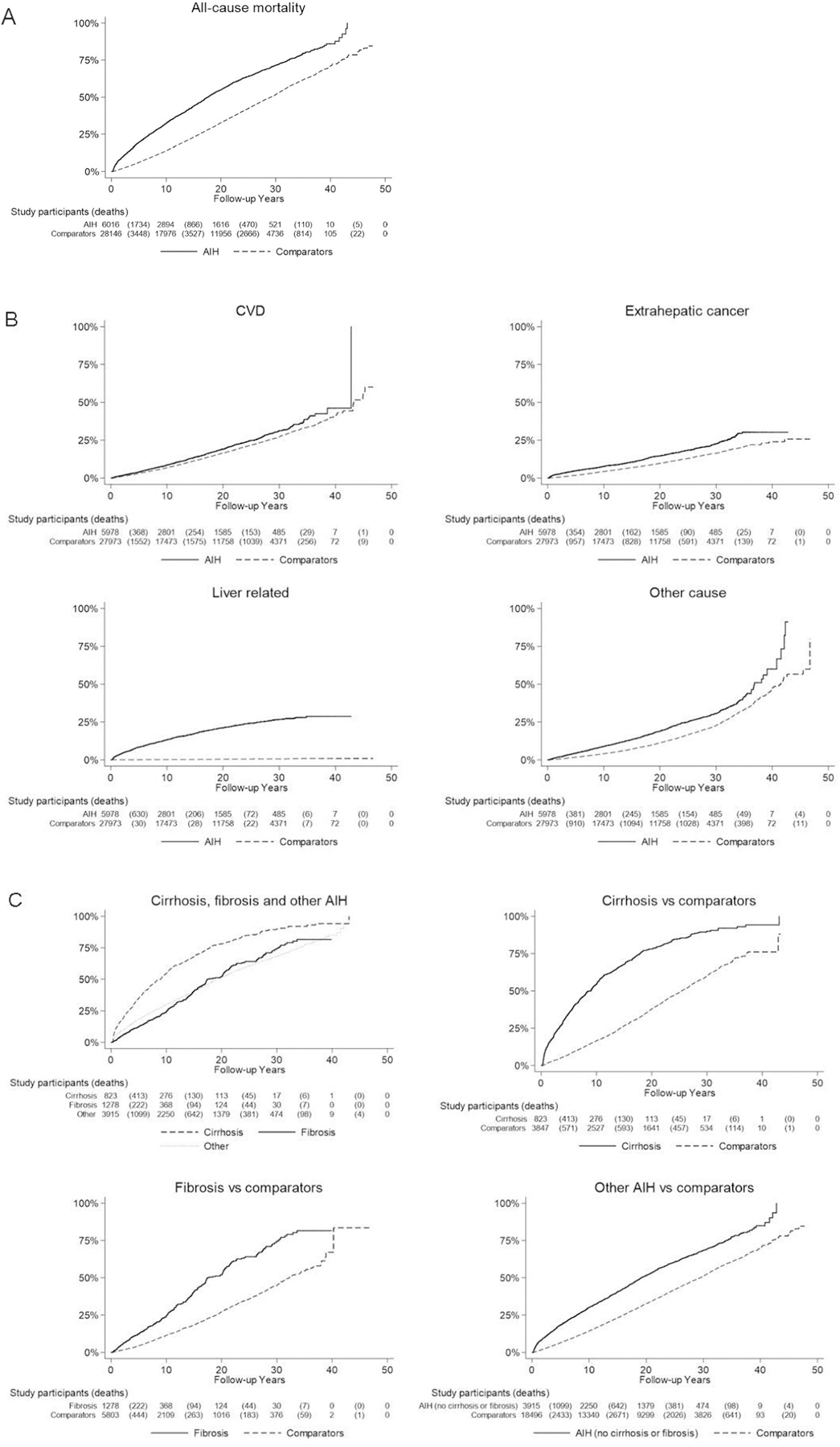
Kaplan-Meier failure curves of time to (*A*) transplant-free all-cause mortality, (*B*) transplant-free cause-specific mortality, and (*C*) transplant-free all-cause mortality by histopathology group.

During follow-up, 3185 (52.9%) individuals with AIH and 10,477 (37.2%) reference individuals either died or underwent liver transplantation (here combined as “death”) (Table 2). Individuals with AIH had a mortality rate of 41.4 (95% CI, 40.0–42.9) per 1000 person-years, compared with 21.9 per 1000 person-years in reference individuals (95% CI, 21.4–22.3) (Table 2). The 10-year cumulative incidence of death was 32.3% (95% CI, 31.1%–33.6%) for AIH individuals and 14.1% (95% CI, 13.7%–14.5%) for reference individuals. In adjusted analyses, individuals with AIH had a 2.29 times higher risk of death compared with reference individuals (95% CI, 2.17–2.41) (Table 2, Figure 2A).

#### Cause-Specific Mortality.

Individuals with AIH not only had a high risk of liver-related death compared with reference individuals (HR, 66.24; 95% CI, 48.19–91.03), but also had a higher risk of CVD (HR, 1.27; 95% CI, 1.15–1.40), extrahepatic cancer (HR, 1.69; 95% CI, 1.51–1.89), and other causes of death (HR, 1.95; 95% CI, 1.77–2.15) (Table 2, Figure 2B).

#### Subanalyses.

During the first year of follow-up (3–<12 months), AIH was associated with a 4.76-fold increased risk of death (95% CI, 3.86–5.86), with the HR then stabilizing around 2 after 5 years’ follow-up and remaining elevated ≥ 20 years’ follow-up (Table 3). HRs were similar in women and men, but were higher in 18- to 29-year-olds than in other age groups. Mortality was similar across calendar periods except for a somewhat lower mortality in individuals diagnosed from 1969 to 1986.

Cirrhosis in AIH was linked to a markedly increased risk of death (HR, 4.55; 95% CI, 3.95–5.25), while inflammation without fibrosis (HR, 2.18; 95% CI, 2.00–2.38), fibrosis (HR, 2.68; 95% CI, 2.29–3.13), and necrosis (HR, 1.98; 95% CI, 1.37–2.85) had a more subtly increased risk of death (Table 3, Figure 2C). AIH individuals with liver failure had a 2.81 times higher risk of death (95% CI, 1.97–4.01), but individuals with portal hypertension had a particularly high mortality (HR, 7.55; 95% CI, 5.71–9.99). An even higher mortality in individuals with AIH and PSC overlap (HR, 107.56; 95% CI, 5.44–2126.51) was observed, while PBC overlap also had a high mortality risk. However, individuals with AIH and diabetes, other autoimmune diseases, or conditions that affect the use of first-line AIH medical treatment only trended toward an increased mortality risk compared with reference individuals.

AIH individuals who had portal hypertension or overlap syndromes had an increased risk of death compared with AIH individuals without (Table 4).

### Sensitivity Analyses

Excluding liver transplants from our primary outcome, the HR was 2.08 (95% CI, 1.98–2.20) (Supplementary Table 5).

When starting follow-up at time of diagnosis ( ≥ 0 months), overall mortality risk in individuals with AIH increased (HR, 2.52; 95% CI, 2.39–2.65), with a HR for death of 14.97 (95% CI, 11.29–19.85) for 0–<3 months (Supplementary Tables 6 and 7).

When restricting our data to study participants without a prior diagnosis of cancer (n = 5433), the HR was 2.26 (95% CI, 2.14–2.39) (Supplementary Table 8). During the ICD-10 era (1997–2017), individuals with newly diagnosed AIH (n = 2248) had a 2.73 times higher risk of death compared with reference individuals (95% CI, 2.39–3.12) (Table 3). Restricting to 5 years’ follow-up did not change the HRs more than marginally for each ICD era (Table 3). The median survival time in individuals with AIH was longest in younger age groups (Supplementary Table 9, Supplementary Figure 1). When restricting to 5 years’ follow-up after diagnosis for each age group, the HR for death continued to be the highest for 18–29 years of age and lowest for ≥ 70 years of age (Table 3). When restricting the time between AIH ICD code and liver biopsy to <1 year, HRs were similar to the main analyses (HR, 2.00; 95% CI, 1.88–2.13) (Supplementary Table 10).

To reduce intrafamilial confounding, we compared 2505 AIH individuals with 4908 first-degree full siblings. Both individuals with AIH and their siblings were on average younger, and had less comorbidities (Supplementary Table 11). Individuals with AIH had a 3.44 times higher mortality risk compared with first-degree siblings (95% CI, 2.97–3.99) (Supplementary Table 12, Supplementary Figure 2).

## Discussion

This is the largest nationwide population-based study to date of more than 6000 individuals with AIH, in which we found a 2-fold increased risk of death even beyond 20 years of follow-up compared with matched reference individuals, with the risk of death highest in persons with cirrhosis.

We confirm findings from a smaller Danish population-based study,^3^ contrary to an improvement in long-term mortality risk reported in smaller studies.^5,7,8,17^ In addition, ours is the first study to use sibling control subjects to reduce intrafamilial confounding from genetic and early environmental factors, which reaffirms that AIH is linked to an increased risk of death. Consistent with earlier studies,^2,5,9^ we found a high risk of liver-related death in AIH but add to the literature by also showing increased CVD and extrahepatic cancer–related death.^3,5,9,18^

In this cohort, 13.7% had cirrhosis at diagnosis, which is lower than the 30% reported in earlier large studies, in which only 76.6%–84% of patients had liver biopsy data.^3,5^ Our reported somewhat lower prevalence of cirrhosis may be due to better representation of the full range of inpatient and outpatient biopsy practices and severity of AIH given that 100% of individuals in this nationwide cohort had liver biopsy data. Prior studies may have missed milder forms of AIH, resulting in higher percentages of severe disease compared with our study.

Approximately one-third of individuals had other pathology changes, of which the most common feature was steatosis, supported by prior results that 17%–30% of individuals with AIH can have steatosis on biopsy.^1^ The second most common feature was normal morphology, but given that we did not have access to patient charts, we could not determine whether these biopsies were performed for remission.

We report an increased risk of death in AIH individuals with cirrhosis on liver biopsy compared with the general population (HR, 4.55),^3,5^ while mortality risks for patients with noncirrhotic fibrosis (HR, 2.68) and inflammation without fibrosis (HR, 2.18) were similar to overall risk. These differences in mortality risk are likely explained by observations that most forms of AIH except very mild disease exhibit fibrosis, while only severe forms exhibit cirrhosis.^1,11^ Furthermore, AIH patients with portal hypertension had an even higher risk of mortality (HR, 7.55), which is expected given that liver decompensation is a strong risk factor for death.^19^

We also show that overlap with PSC or PBC has increased mortality,^20^ but this risk may be overestimated due to small numbers. Our cohort contained a lower proportion of females than earlier cohorts (for instance 60.6% vs. 72.0% in Grønbæk et al)^3^; however, mortality did not differ between men and women, and hence, the sex distribution is unlikely to have affected our main results. Younger age groups had higher mortality risk likely due to low death rates in younger reference individuals. Finally, individuals with AIH who require second-line medications do not have increased mortality risk, but given that we could not directly compare first-line with second-line medications, do not have information on hospital-administered medications such as rituximab and biologics, and do not know indications for medications, it remains unknown whether second-line medications are beneficial in certain AIH individuals and future studies are required.^1,11^ Similar to a prior population-based study from Sweden, our study showed that 28.5% were not on any medication.^5^ Our nationwide study may include some very mild cases as opposed to smaller studies at tertiary care centers, and thus we would expect slightly higher proportions of individuals off therapy.

This study has a number of strengths. This is the largest population-based study to date with 6016 patients, and 3185 deaths over complete follow-up, giving this study exceptional power to determine not only overall mortality but also mortality in key subgroups. Given that guidelines require liver biopsy for AIH diagnosis,^1,11^ the addition of liver biopsy to ICD code reduces misclassification and allows us to more accurately capture the most AIH individuals compared with any other large population-based study. In addition, linkage to Swedish Registers allows for nearly 100% follow-up.^14^ We excluded 0–<3 months follow-up to reduce misclassification of biopsies performed for autopsy or in terminally ill patients, and therefore, increase the robustness of our mortality risk estimation. Finally, this is the first population-based study to include sibling comparisons that allow for the minimization of intrafamilial confounding.

This study has some limitations. We did not have access to patient biological samples, charts, or laboratory tests to confirm diagnoses, track disease activity, and determine response to treatments. Few individuals had repeat liver biopsies. In this cohort, we report a 96% PPV (95% CI, 89%–99%) of having a histopathology report consistent with AIH indicating high accuracy of our AIH criteria, and show that a high proportion (82%) have 2 AIH ICD codes, further supporting diagnostic accuracy. Furthermore, we analyzed mortality risk in patients diagnosed with AIH since 2002 to coincide with the introduction of a revised scoring system for AIH,^1,11^ the introduction of ICD-10, the introduction of hepatitis C virus testing, and the inclusion of hospital-based outpatient data, which in general produced similar estimates as the main analysis. Restricting the time between ICD code and liver biopsy to within 1 year to confirm that liver biopsy was performed as part of AIH workup resulted in similar HR. We cannot rule out some selection bias, given that biopsy practices in the past may have varied and that patients at high risk for biopsy may not have been biopsied. To address potential confounding by comorbidities, even after adjusting for the higher prevalence of CVD, diabetes, and cancer, the positive association between AIH and death still persisted, suggesting that the increased risk of mortality in AIH is not confounded by comorbidities. Finally, we cannot rule out residual confounding, as the National Registers do not have reliable data on parameters such as smoking, obesity, or race or ethnicity.

In conclusion, this nationwide population-based study with complete liver biopsy data found a 2-fold increased risk of death in AIH, a risk that persisted beyond 20 years’ follow-up. Concomitant cirrhosis, portal hypertension, and overlap syndromes with PBC or PSC were risk factors for death and these patients should be followed closely.

## Supplementary Material

1

## Figures and Tables

**Table 1.** Characteristics of Individuals With AIH and Reference Individuals

Group	AIH Individuals (n = 6016)	Reference Individuals (n = 28,146)
Follow-up		
Mean (SD)	12.8 (10.3)	17.0 (11.0)
Median (IQR)	9.5 (4.2–21.0)	15.8 (6.9–26.3)
Range (minimum–maximum)	0.3–43.1	0.3–47.8
Follow-up category		
3–<12 mo	6016 (100)	28,146 (100)
1–<5 y	5647 (93.9)	27,859 (99.0)
5–<10 y	4220 (70.1)	23,292 (82.8)
≥10 y	2894 (48.1)	17,976 (63.9)
≥20 y	1616 (26.9)	11,956 (42.5)
≥30 y	521 (8.7)	4736 (16.8)
Sex		
Female	3644 (60.6)	17,134 (60.9)
Male	2372 (39.4)	11,012 (39.1)
Age, y		
Mean (SD)	53.5 (16.3)	53.4 (16.1)
Median (IQR)	55.2 (41.1–66.4)	55.1 (41.4–66.1)
Range (minimum–maximum)	18.1–93.5	18.0–93.5
Age category		
18y –<30 y	615 (10.2)	2825 (10.0)
30–<40 y	775 (12.9)	3573 (12.7)
40–<50 y	992 (16.5)	4736 (16.8)
50–<60 y	1254 (20.8)	5991 (21.3)
60–<70 y	1354 (22.5)	6422 (22.8)
≥70 y	1026 (17.1)	4599 (16.3)
Year of diagnosis		
1969–1986	2019 (33.6)	9802 (34.8)
1987–2001	1974 (32.8)	9307 (33.1)
2002–2017	2023 (33.6)	9037 (32.1)
Time to register-based definition of AIH onset (time in years between first AIH diagnosis and biopsy)		
Mean (SD)	2.9 (5.7)	
Median (IQR)	0.2 (0.0–3.0)	
Range (minimum–maximum)	0.0–45.5	
Country of birth		
Nordic	5673 (94.3)	26,411 (93.8)
Other	341 (5.7)	1733 (6.2)
Missing	2 (0.0)	2 (0.0)
Level of education		
≤9 y	1902 (31.6)	9757 (34.7)
10–12 y	2161 (35.9)	10,192 (36.2)
>12 y	1167 (19.4)	6014 (21.4)
Missing	786 (13.1)	2183 (7.8)
Level of education using highest level of education in parents when missing		
≤9 y	1937 (32.2)	9785 (34.8)
10–12 y	2171 (36.1)	10,213 (36.3)
>12 y	1177 (19.6)	6022 (21.4)
Missing	731 (12.2)	2126 (7.6)
Pathology findings		
Cirrhosis (stage F4)	823 (13.7)	
Fibrosis (stage F1–F3)	1278 (21.2)	
Inflammation without fibrosis (stage F0)	2068 (34.4)	
Other or unspecified pathology findings	1847 (30.7)	
Necrosis	148 (2.5)	
Severity of liver disease^a^		
Portal hypertension	294 (4.9)	
Liver failure	157 (2.6)	
AIH treatment during follow-up		
Second-line medications	216 (12.2)	
Azathioprine and not prednisolone/prednisone	370 (21.0)	
Prednisolone/prednisone and not azathioprine	153 (8.7)	
Both azathioprine and prednisolone/prednisone	283 (16.0)	
Oral budesonide	240 (13.6)	
Oral budesonide	240 (13.6)	
Overlap syndromes^a^		
Primary sclerosing cholangitis	92 (1.5)	
Primary biliary cholangitis	247 (4.1)	
Comorbidities^b^		
Cardiovascular disease	829 (13.8)	1710 (6.1)
Extrahepatic malignancy	422 (7.0)	822 (2.9)
Diabetes	612 (10.2)	594 (2.1)
End-stage renal disease	13 (0.2)	24 (0.1)
Other autoimmune diseases	874 (14.5)	474 (1.7)
Presence of conditions that affect use of first-line medical treatment	488 (8.1)	801 (2.8)

AIH, autoimmune hepatitis; IQR, interquartile range; SD, standard deviation.

a At any time point prior to AIH diagnosis.

b Within the past 5 years prior to AIH diagnosis.

**Table 2.** Risk of All-Cause and Cause-Specific Mortality in Individuals With AIH and Reference Individuals

	All-cause mortality		Cardiovascular disease		Extrahepatic cancer		Liver related		Other cause	
	AIH	Reference	AIH	Reference	AIH	Reference	AIH	Reference	AIH	Reference
N	6016	28,146	5978	27,973	5978	27,973	5978	27,973	5978	27,973
Death or liver transplantation	3185 (52.9)	10,477 (37.2)	805 (13.5)	4431 (15.8)	631 (10.6)	2516 (9.0)	914 (15.3)	87 (0.3)	833 (13.9)	3441 (12.3)
Death	2952 (49.1)	10,473 (37.2)	805 (13.5)	4431 (15.8)	631 (10.6)	2516 (9.0)	681 (11.4)	83 (0.3)	833 (13.9)	3441 (12.3)
Liver transplantation	233 (3.9)	4 (0.0)	0 (0.0)	0 (0.0)	0 (0.0)	0 (0.0)	233 (3.9)	4 (0.0)	0 (0.0)	0 (0.0)
Follow-up, y										
Mean (SD)	12.8 (10.3)	17.0 (11.0)	12.4 (10.2)	16.5 (10.9)	12.4 (10.2)	16.5 (10.9)	12.4 (10.2)	16.5 (10.9)	12.4 (10.2)	16.5 (10.9)
Median (IQR)	9.5 (4.2–21.0)	15.8 (6.9–26.3)	9.1 (3.8–20.6)	15.6 (6.3–25.8)	9.1 (3.8–20.6)	15.6 (6.3–25.8)	9.1 (3.8–20.6)	15.6 (6.3–25.8)	9.1 (3.8–20.6)	15.6 (6.3–25.8)
Range (minimum–maximum)	0.3–43.1	0.3–47.8	0.3–42.8	0.3–46.8	0.3–42.8	0.3–46.8	0.3–42.8	0.3–46.8	0.3–42.8	0.3–46.8
Incidence rate per 1000 PY (95% CI)	41.4 (40.0–42.9)	21.9 (21.4–22.3)	10.9 (10.1–11.6)	9.6 (10.1–11.6)	8.5 (7.9–9.2)	5.4 (5.2–5.7)	12.3 (11.5–13.1)	0.2 (0.1–0.2)	11.2 (10.5–12.0)	7.4 (7.2–7.7)
HR (95% CI)										
Conditioned^a^	2.86 (2.72–2.99)		1.66 (1.52–1.81)		2.03 (1.84–2.24)		77.56 (58.49–102.87)		2.38 (2.18–2.61)	
Adjusted^b^	2.29 (2.17–2.41)		1.27 (1.15–1.40)		1.69 (1.51–1.89)		66.24 (48.19–91.03)		1.95 (1.77–2.15)	

NOTE. Values are n (%), unless otherwise indicated.

AIH, autoimmune hepatitis; CI, confidence interval; HR, hazard ratio; IQR, interquartile range; PY, person-years.

a Conditioned on age, sex, county, and calendar period.

b Conditioned and further adjusted for education and baseline medical comorbidities and factors that would preclude treatment with first-line therapy.

**Table 3.** Risk of All-Cause Mortality in Subgroups of Individuals With AIH and Reference Individuals

Group	Patients		Events		Incidence Rate (95% CI) per 1000 PY		HR (95%CI)^a^	HR (95%CI)^b^
	AIH	Reference	AIH	Reference	AIH	Reference		
Overall (≥3 mo)	6016 (100)	28,146 (100)	3185 (52.9)	10,477 (37.2)	41.4 (40.0–42.9)	21.9 (21.4–22.3)	2.86 (2.72–2.99)	2.29 (2.17–2.41)
Follow-up								
3–<12 mo	6016 (100)	28,146 (100)	364 (6.1)	244 (0.9)	7.0 (6.3–7.7)	1.0 (0.8–1.1)	7.74 (6.54–9.17)	4.76 (3.86–5.86)
1–<5y	5647 (93.9)	27,859 (99.0)	757 (13.4)	1408 (5.1)	38.4 (35.7–41.2)	13.7 (13.0–14.4)	3.15 (2.87–3.47)	2.45 (2.19–2.75)
5–<10 y	4220 (70.1)	23,292 (82.8)	613 (14.5)	1796 (7.7)	34.9 (32.1–37.6)	17.5 (16.7–18.3)	2.58 (2.32–2.86)	2.05 (1.82–2.31)
≥10 y	2894 (48.1)	17,976 (63.9)	1451 (50.1)	7029 (39.1)	43.0 (40.8–45.2)	28.6 (27.9–29.2)	2.37 (2.21–2.54)	2.11 (1.96–2.28)
≥20 y	1616 (26.9)	11,956 (42.5)	585 (36.2)	3502 (29.3)	48.5 (44.6–52.5)	35.1 (34.0–36.3)	2.20 (1.97–2.46)	2.04 (1.81–2.29)
≥30 y	521 (8.7)	4736 (16.8)	115 (22.1)	836 (17.7)	63.5 (51.9–75.1)	47.9 (44.7–51.1)	1.80 (1.40–2.32)	1.73 (1.32–2.25)
≥1 y	5647 (93.9)	27,859 (99.0)	2821 (50.0)	10,233 (36.7)	39.7 (38.3–41.2)	22.7 (22.2–23.1)	2.61 (2.48–2.74)	2.17 (2.05–2.29)
Sex								
Female	3644 (60.6)	17,134 (60.9)	1827 (50.1)	6012 (35.1)	41.4 (39.5–43.3)	21.9 (21.3–22.4)	2.84 (2.67–3.02)	2.34 (2.18–2.50)
Male	2372 (39.4)	11012 (39.1)	1358 (57.3)	4465 (40.5)	41.4 (39.2–43.7)	21.8 (21.2–22.5)	2.88 (2.68–3.10)	2.22 (2.04–2.41)
Age at diagnosis, all follow-up								
18–<30 y	615 (10.2)	2825 (10.0)	115 (18.7)	79 (2.8)	10.9 (8.9–12.9)	1.4 (1.1–1.7)	8.10 (5.93–11.07)	7.11 (4.94–10.25)
30–<40 y	775 (12.9)	3573 (12.7)	257 (33.2)	283 (7.9)	18.4 (16.2–20.7)	3.5 (3.1–3.9)	6.15 (5.07–7.47)	4.59 (3.71–5.69)
40–<50 y	992 (16.5)	4736 (16.8)	434 (43.8)	915 (19.3)	25.9 (23.5–28.3)	8.8 (8.3–9.4)	3.52 (3.09–4.01)	2.84 (2.45–3.30)
50–<60 y	1254 (20.8)	5991 (21.3)	700 (55.8)	2293 (38.3)	40.8 (37.7–43.8)	20.5 (19.7–21.3)	2.74 (2.48–3.02)	2.10 (1.88–2.35)
60–<70 y	1354 (22.5)	6422 (22.8)	882 (65.1)	3597 (56.0)	70.5 (65.9–75.2)	41.6 (40.3–43.0)	2.50 (2.29–2.74)	1.96 (1.77–2.17)
≥70 y	1026 (17.1)	4599 (16.3)	797 (77.7)	3310 (72.0)	134.3 (124.9–143.6)	79.4 (76.7–82.1)	2.30 (2.10–2.53)	1.83 (1.65–2.03)
Age at diagnosis, restricting follow-up to 5 y								
18–<30 y	615 (10.2)	2825 (10.0)	26 (4.2)	12 (0.4)	9.2 (5.7–12.7)	0.9 (0.4–1.4)	9.65 (4.86–19.18)	13.24 (4.56–38.46)
30–<40 y	775 (12.9)	3573 (12.7)	61 (7.9)	19 (0.5)	17.1 (12.8–21.4)	1.1 (0.6–1.6)	16.74 (9.64–29.07)	10.27 (5.12–20.63)
40–<50 y	992 (16.5)	4736 (16.8)	88 (8.9)	53 (1.1)	19.5 (15.4–23.5)	2.3 (1.7–3.0)	8.38 (5.86–11.96)	9.73 (5.32–17.79)
50–<60 y	1254 (20.8)	5991 (21.3)	170 (13.6)	178 (3.0)	30.7 (26.1–35.3)	6.3 (5.4–7.2)	4.95 (3.98–6.15)	3.91 (2.80–5.46)
60–<70 y	1354 (22.5)	6422 (22.8)	321 (23.7)	458 (7.1)	58.2 (51.8–64.5)	15.5 (14.1–16.9)	3.95 (3.40–4.59)	2.63 (2.14–3.23)
≥70 y	1026 (17.1)	4599 (16.3)	455 (44.3)	932 (20.3)	127.3 (115.6–139.0)	47.2 (44.2–50.3)	2.95 (2.61–3.34)	2.31 (2.02–2.64)
Year of diagnosis								
1969–1986	2019 (33.6)	9802 (34.8)	1616 (80.0)	6073 (62.0)	47.2 (44.9–49.5)	26.1 (25.5–26.8)	2.71 (2.54–2.90)	2.08 (1.93–2.25)
1987–2001	1974 (32.8)	9307 (33.1)	1212 (61.4)	3706 (39.8)	40.0 (37.7–42.2)	19.9 (19.3–20.6)	3.00 (2.78–3.25)	2.41 (2.21–2.62)
2002–2017	2023 (33.6)	9037 (32.1)	357 (17.6)	698 (7.7)	28.9 (25.9–32.0)	11.4 (10.6–12.3)	3.04 (2.64–3.50)	2.67 (2.29–3.11)
1997–2017	2248 (37.4)	10 063 (35.8)	468 (20.8)	980 (9.7)	30.9 (28.1–33.7)	12.6 (11.8–13.4)	3.14 (2.77–3.55)	2.73 (2.39–3.12)
Year of diagnosis, restricting follow-up to 5 y								
1969–1986	2019 (33.6)	9802 (34.8)	444 (22.0)	721 (7.4)	50.4 (45.7–55.1)	15.3 (14.2–16.4)	3.69 (3.26–4.19)	2.06 (1.72–2.47)
1987–2001	1974 (32.8)	9307 (33.1)	413 (20.9)	556 (6.0)	47.7 (43.1–52.3)	12.3 (11.3–13.4)	4.42 (3.85–5.07)	3.24 (2.76–3.81)
2002–2012	1333 (22.2)	5957 (21.2)	216 (16.2)	301 (5.1)	36.0 (31.2–40.8)	10.4 (9.2–11.6)	3.95 (3.27–4.77)	3.48 (2.82–4.28)
Country of birth								
Nordic	5673 (94.3)	26,411 (93.8)	3032 (53.4)	10,114 (38.3)	41.6 (40.2–43.1)	22.2 (21.8–22.7)	2.83 (2.70–2.97)	2.26 (2.14–2.38)
Other	341 (5.7)	1733 (6.2)	152 (44.6)	363 (20.9)	37.7 (31.7–43.7)	15.0 (13.5–16.5)	4.93 (2.36–10.31)	2.41 (0.96–6.02)
Education								
≤9 y	1937 (32.2)	9785 (34.8)	1307 (67.5)	5177 (52.9)	47.7 (45.1–50.3)	28.4 (27.6–29.2)	2.54 (2.32–2.77)	2.10 (1.91–2.31)
10–12 y	2171 (36.1)	10,213 (36.3)	843 (38.8)	2382 (23.3)	26.9 (25.0–28.7)	13.3 (12.8–13.9)	3.35 (2.93–3.82)	2.91 (2.54–3.35)
>12 y	1177 (19.6)	6022 (21.4)	317 (26.9)	861 (14.3)	21.6 (19.2–23.9)	8.5 (7.9–9.1)	4.06 (3.05–5.38)	3.16 (2.31–4.31)
Missing	731 (12.2)	2126 (7.6)	718 (98.2)	2057 (96.8)	213.8 (198.1–229.4)	121.7 (116.4–126.9)	1.94 (1.69–2.22)	1.57 (1.35–1.82)
Pathology findings								
Cirrhosis (stage F4)	823 (13.7)	3847 (13.7)	594 (72.2)	1736 (45.1)	79.5 (73.1–85.9)	26.5 (25.3–27.8)	5.75 (5.07–6.52)	4.55 (3.95–5.25)
Fibrosis (stage F1–F3)	1278 (21.2)	5803 (20.6)	367 (28.7)	950 (16.4)	33.5 (30.1–37.0)	15.3 (14.3–16.2)	3.07 (2.67–3.54)	2.68 (2.29–3.13)
Inflammation without Fibrosis (stage F0)	2068 (34.4)	9720 (34.5)	1096 (53.0)	3690 (38.0)	38.3 (36.1–40.6)	21.3 (20.6–22.0)	2.68 (2.47–2.90)	2.18 (2.00–2.38)
Other or unspecified pathology findings	1847 (30.7)	8776 (31.2)	1128 (61.1)	4101 (46.7)	37.8 (35.6–40.0)	23.0 (22.3–23.7)	2.28 (2.11–2.47)	1.77 (1.63–1.93)
Necrosis	148 (2.5)	684 (2.4)	66 (44.6)	209 (30.6)	31.6 (24.0–39.3)	17.8 (15.4–20.2)	2.02 (1.48–2.77)	1.98 (1.37–2.85)
Severity of liver disease								
Portal hypertension	294 (4.9)	1363 (4.8)	225 (76.5)	554 (40.6)	129.3 (112.4–146.2)	27.5 (25.2–29.8)	9.76 (7.68–12.40)	7.55 (5.71–9.99)
Liver failure	157 (2.6)	744 (2.6)	84 (53.5)	254 (34.1)	41.1 (32.3–49.9)	18.5 (16.3–20.8)	3.79 (2.79–5.14)	2.81 (1.97–4.01)
Overlap syndromes								
Primary sclerosing cholangitis	92 (1.5)	415 (1.5)	46 (50.0)	31 (7.5)	83.2 (59.1–107.2)	6.4 (4.1–8.6)	23.89 (11.25–50.74)	107.56 (5.44–2126.51)
Primary biliary cholangitis	247 (4.1)	1121 (4.0)	105 (42.5)	219 (19.5)	64.4 (52.1–76.8)	18.2 (15.8–20.7)	6.29 (4.63–8.54)	5.88 (4.22–8.21)
Comorbidities								
Cardiovascular disease	829 (13.8)	1710 (6.1)	599 (72.3)	1036 (60.6)	104.1 (95.8–112.4)	71.8 (67.5–76.2)	1.61 (1.29–2.02)	1.49 (1.17–1.89)
Malignancy	422 (7.0)	822 (2.9)	301 (71.3)	397 (48.3)	109.6 (97.2–122.0)	57.5 (51.9–63.2)	2.21 (1.18–4.13)	2.18 (1.06–4.49)
Diabetes	612 (10.2)	594 (2.1)	436 (71.2)	320 (53.9)	89.0 (80.6–97.3)	73.5 (65.4–81.5)	1.51 (0.93–2.44)	1.78 (0.98–3.25)
End-stage renal disease	13 (0.2)	24 (0.1)	13 (100.0)	10 (41.7)	124.7 (56.9–192.4)	74.6 (28.4–120.9)	—	—
Other autoimmune diseases	874 (14.5)	474 (1.7)	323 (37.0)	143 (30.2)	40.8 (36.4–45.3)	37.5 (31.4–43.6)	2.24 (1.08–4.65)	2.45 (0.99–6.04)
Presence of conditions that affect use of first-line medical treatment	488 (8.1)	801 (2.8)	264 (54.1)	180 (22.5)	48.6 (42.7–54.5)	14.6 (12.5–16.8)	3.66 (1.46–9.15)	4.64 (1.01–21.21)

NOTE. Values are n (%), unless otherwise indicated.

AIH, autoimmune hepatitis; CI, confidence interval; HR, hazard ratio; PY, person-years.

a Conditioned on age, sex, county, and calendar period.

b Conditioned and further adjusted for education and baseline medical comorbidities and factors that would preclude treatment with first-line therapy.

**Table 4.** Risk of All-Cause Mortality in Individuals With AIH

Group	Patients		Events		Incidence Rate (95% CI) per 1000 PY		HR (95%CI)^a^	HR (95%CI)^b^
	AIH + factor	AIH no factor	AIH + Factor	AIH No Factor	AIH + Factor	AIH No Factor		
AIH with necrosis vs no necrosis	148	5868	66(44.6)	3119 (53.2)	31.6 (24.0–39.3)	41.7 (40.3–43.2)	0.78 (0.61–0.99)	0.68 (0.53–0.87)
AIH with PSC and/or PBC vs no PSC and/or PBC	331	5685	148 (44.7)	3037 (53.4)	68.8 (57.7–79.9)	40.7 (39.2–42.1)	2.27 (1.92–2.70)	2.35 (1.97–2.79)
AIH with portal hypertension vs no portal hypertension	294	5722	225 (76.5)	2960 (51.7)	129.3 (112.4–146.2)	39.4 (38.0–40.8)	3.07 (2.68–3.53)	2.87 (2.50–3.30)
AIH using second-line medication vs no second-line medication (time-dependent analysis)	—	—	—	—	—	—	1.23 (0.83–1.81)	1.23 (0.83–1.83)

NOTE. Values are n or n (%), unless otherwise indicated.

AIH, autoimmune hepatitis; CI, confidence interval; HR, hazard ratio; PBC, primary biliary cholangitis; PSC, primary sclerosing cholangitis; PY, person-years.

a Adjusted for age, sex, and year of diagnosis.

b Adjusted for age, sex, year of diagnosis, education, and baseline medical comorbidities and factors that would preclude treatment with first-line therapy.

## What You Need to Know

### Background

The prognosis of autoimmune hepatitis (AIH) patients compared with the general population remains unclear. Prior studies include small single-center studies comprising sicker patients or population-based studies only using diagnosis codes.

### Findings

This is the largest population-based study with complete liver biopsy data. We show a 2 times higher risk of death in AIH patients compared with the general population.

### Implications for patient care

These results provide physicians with accurate information on the overall prognosis of AIH patients and key subgroups including fibrosis status in order to appropriately monitor and counsel high-risk patients.

## Abbreviations used in this paper:

AIH: autoimmune hepatitis
CVD: cardiovascular disease
CI: confidence interval
HR: hazard ratio
ICD: International Classification of Diseases
ICD-10: International Classification of Diseases–Tenth Revision
PSC: primary sclerosing cholangitis
PBC: primary biliary cholangitis
