# Supplementary material for: Increased Mortality Risk in Autoimmune Hepatitis: A Nationwide Population-Based Cohort Study With Histopathology
Source: Clin Gastroenterol Hepatol. Author manuscript; Available in PMC 2022 Dec 1. (PMC9347643; doi:10.1016/j.cgh.2020.10.006)
Supplement: 1 [file NIHMS1638413-supplement-1.docx]

**Supplementary Material**


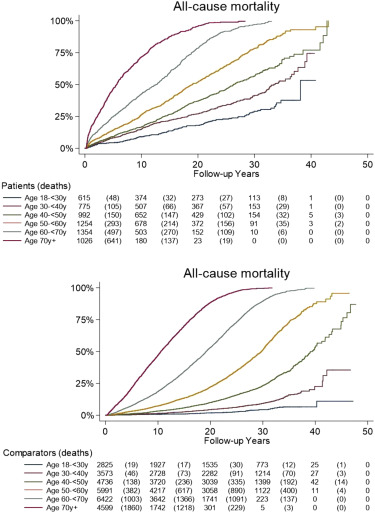


Supplementary Figure 1. Kaplan-Meier failure curves of time to transplant-free all-cause mortality (follow-up until December 31, 2017) by age in patients with autoimmune hepatitis (AIH) (upper panel) and in matched general population comparators (lower panel).


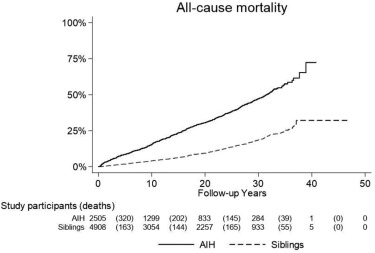


Supplementary Figure 2. Kaplan-Meier failure curves of time to transplant-free all-cause mortality for individuals with autoimmune hepatitis (AIH) and siblings.

Supplementary Table 1. [ICD](https://www.sciencedirect.com/topics/medicine-and-dentistry/international-classification-of-diseases) Codes for Autoimmune Hepatitis That Were Included and ICD Codes for Other [Chronic Liver Diseases](https://www.sciencedirect.com/topics/medicine-and-dentistry/chronic-liver-disease) That Were Excluded Prior to or at the Start of Follow-Up

| **Diagnosis** | **ICD-8 (1969–1986)** | **ICD-9 (1987–1996)** | **ICD-10 (1997–)** |
| --- | --- | --- | --- |
| Autoimmune hepatitis | 573,0x; 571.9x | 573D; 571E | K75.4 |
| Viral hepatitis | 070 999.20 | 070 | B15–B19 B00.8 (HSV hepatitis) B25.1 (CMV hepatitis) B24 (HIV) K77.0 (infectious hepatitis) |
| Hereditary hemochromatosis | 273.20 | 275A | E83.1 |
| Alpha 1 Anti-trypsin deficiency | — | 274E 277G | E88.0X |
| Wilson’s disease | 273.30 | 275B | E83.0 |
| Drug induced liver injury | — | 573W | K71.x |
| Alcohol liver disease or alcohol use disorders | 571.00 571.01 571.09 258, 291.1, 299 | 571A 571B 571C 571D 255, 294A, 291, 303, 305A, 357F, 425F, 535D, 655E, 980 | K70.x F10.x, E24.4, F04.9, G31.2, G62.1, G72.1, I42.6, K29.2, K85.2, K86.0, O35.4, X65, Y15, Y91 |
| Nonalcoholic fatty liver disease | 571.01 | 571W | K75.8 K76.0 |
| Congestive hepatopathy |  | 573A | K76.1 |
| Budd-Chiari | 453.01 | 453A | I82.0, K76.5 |

ICD, International Classification of Diseases; ICD, International Classification of Diseases–Eighth Revision; ICD-9, International Classification of Diseases–Ninth Revision; ICD-10, International Classification of Diseases–Tenth Revision.

Supplementary Table 2. SNOMED Codes for Liver [Histopathology](https://www.sciencedirect.com/topics/medicine-and-dentistry/histopathology)

| **Histopathology subtype** | **SNOMED code** |
| --- | --- |
| Cirrhosis (stage F4) | M4950x |
| Fibrosis (stage F1–F3) | M4500x M4900xx |
| Inflammation without fibrosis (stage F0) | M4xxxx inflammation codes minus codes above for cirrhosis and fibrosis |
| Other (includes normal liver biopsy) | M001xx and all other codes present that are not listed above |
| Necrosis | M40700 M41700 M540xx–M541xx |

SNOMED, [Systematized Nomenclature of Medicine](https://www.sciencedirect.com/topics/medicine-and-dentistry/systematized-nomenclature-of-medicine).

Supplementary Table 3. [ICD](https://www.sciencedirect.com/topics/medicine-and-dentistry/international-classification-of-diseases) Codes for Cause-Specific Death

| **Cause-Specific Death** | **ICD-8 (1969–1986)** | **ICD-9 (1987–1996)** | **ICD-10 (1997–)** |
| --- | --- | --- | --- |
| Cardiovascular | 391–458 | 391–459 | I01–I99 |
| Extrahepatic cancer | 140–209, 235–239; exclude 155.0 | 140–209, 235–239; exclude 155A | C00–C99; exclude C22.0 |
| Liver-related death, including |  |  |  |
| Liver failure | 570 | 570 | K72.0 |
| HCC | 155.0 | 155A | C22.0 |
| Liver transplantation | 5200–5299 | 5200–5299 | JJC surgery codes Z94.4 |
| Liver disease | 570–576 | 571–573 | K70–K77 |
| Other | Any remaining deaths were placed in this category | — | — |

HCC, hepatocellular carcinoma; ICD, International Classification of Diseases; ICD, International Classification of Diseases–Eighth Revision; ICD-9, International Classification of Diseases–Ninth Revision; ICD-10, International Classification of Diseases–Tenth Revision.

Supplementary Table 4. [ICD](https://www.sciencedirect.com/topics/medicine-and-dentistry/international-classification-of-diseases) Codes for Covariates

| **Variable** | **ICD-8 (1969–1986)** | **ICD-9 (1987–1996)** | **ICD-10 (1997–)** |  |
| --- | --- | --- | --- | --- |
| Portal hypertension (includes any of the following codes as 1 grouped variable; of note, there are ICD codes available for hepatic encephalopathy)[^a^](https://www.sciencedirect.com/science/article/pii/S1542356520313951?via%3Dihub#tblS4fna) | | | | |
| Esophageal varices (presence of varices at endoscopy or presence of bleeding varices) | 456 | 456A, 456B, 456C | I85.0, I85.9 | |
| Ascites | 785.3 | 789F | R18.9 | |
| Hepatorenal syndrome | — | 572E | K76.7 | |
| Liver failure[^a^](https://www.sciencedirect.com/science/article/pii/S1542356520313951?via%3Dihub#tblS4fna) | | | |  |
| Acute/subacute liver failure (does not include codes for chronic liver failure or unspecified liver failure) | 570 | 570 | K72.0 | |
| Overlap syndromes[^a^](https://www.sciencedirect.com/science/article/pii/S1542356520313951?via%3Dihub#tblS4fna) | | | |  |
| PSC | 575.05 + 563 | 5761 + either of 555 or 556 | K83.0A + code for IBD (K50 or K51) | |
| PBC | — | 571G | K74.3, K74.5 | |
| Other autoimmune disease (includes any of the following codes as 1 grouped variable)[^b^](https://www.sciencedirect.com/science/article/pii/S1542356520313951?via%3Dihub#tblS4fnb) | | | |  |
| Autoimmune thyroid disease | 242.00, 242.09, 244 245.03 | 242A, 242X, 244X, 245C, 245W | E03.5, E03.9, E05.0, E05.5, E05.9, E06.3, E06.5 | |
| Type 1 diabetes | 250 Restricted to individuals with a first ICD-code at age 30 y or below (<31 y). | 250 Restricted to individuals with a first ICD-code at age 30 y or below (<31 y). | E10 | |
| Ulcerative colitis | 563.19 | 556 | K51 | |
| Crohn’s disease | 563.10 | 555 | K50 | |
| Celiac disease | 269.00 | 579A | K90.0 | |
| Vitiligo | 709.01 | 709A | L80 | |
| Rheumatoid arthritis | 712.19, 712.39, 712.59 | 714 | M05, M06, M12.3 | |
| SLE | 734.1 | 710A | M32 | |
| Presence of factors that affect use of first-line medical treatment (includes any of the following codes as 1 grouped variable)[^b^](https://www.sciencedirect.com/science/article/pii/S1542356520313951?via%3Dihub#tblS4fnb) | | | |  |
| Any infection | 001–136 | 001–136 | A00–A99 B00–B99 | |
| Tuberculosis | 010–019 | 010–018 | A15–A19 | |
| Psychosis | 295–299 | 291–299 | F20–F29 | |
| Lymphoma | 200–202 | 200–202 | C81–C88 | |
| Vertebral compression fracture | 805, 806 | 805, 806 | S12, S220, S221, S32.0, S32.9 | |
| Comorbidities[^b^](https://www.sciencedirect.com/science/article/pii/S1542356520313951?via%3Dihub#tblS4fnb) | | | |  |
| Cardiovascular disease | 393–398 410–449 | 393–398, 410–449 | I05–I09, I20–I79 | |
| Extrahepatic cancer | 140–209, 235–239; Exclude 155.0 | 140–209, 235–239; exclude 155A | C00–C99; exclude C22.0 | |
| Diabetes | 250 | 250 | E10–E14 | |
| ESRD grouped as 1 variable |  |  |  | |
| Medical diagnosis of ESRD | — | 585 | N18.0; N18.5 | |
| Renal dialysis | Y29.01 | V45B; V56 | Z49; Z99.2 | |
| Renal transplantation |  | V42A | Z94.0 | |

[ESRD](https://www.sciencedirect.com/topics/medicine-and-dentistry/end-stage-renal-disease), end-stage renal disease; ICD, International Classification of Diseases; ICD, International Classification of Diseases–Eighth Revision; ICD-9, International Classification of Diseases–Ninth Revision; ICD-10, International Classification of Diseases–Tenth Revision; [PBC](https://www.sciencedirect.com/topics/medicine-and-dentistry/primary-biliary-cirrhosis), primary biliary [cirrhosis](https://www.sciencedirect.com/topics/medicine-and-dentistry/liver-cirrhosis); [PSC](https://www.sciencedirect.com/topics/medicine-and-dentistry/primary-sclerosing-cholangitis) primary sclerosing cirrhosis; [SLE](https://www.sciencedirect.com/topics/medicine-and-dentistry/systemic-lupus-erythematosus), systemic lupus erythematosus.

a

At any time point prior to autoimmune hepatitis diagnosis.

b

Within the past 5 years prior to autoimmune hepatitis diagnosis.

Supplementary Table 5. Risk of All-Cause Mortality in Patients With AIH and Matched General Population Comparators: Mortality Only

| **Group** | **Patients** | | **Events** | | **Incidence rate (95% CI) per 1000 PY** | | **HR (95% CI)****^[a](https://www.sciencedirect.com/science/article/pii/S1542356520313951?via%3Dihub#tblS5fna)^** | **HR (95% CI)****^[b](https://www.sciencedirect.com/science/article/pii/S1542356520313951?via%3Dihub#tblS5fnb)^** |
| --- | --- | --- | --- | --- | --- | --- | --- | --- |
|  | **AIH** | **Comparators** | **AIH** | **Comparators** | **AIH** | **Comparators** |  |  |
| Overall (≥3 mo) | 6016 (100) | 28,146 (100) | 2952 (49.1) | 10,473 (37.2) | 38.4 (37.0–39.8) | 21.8 (21.4–22.3) | 2.62 (2.50–2.75) | 2.08 (1.98–2.20) |
| Follow-up |  |  |  |  |  |  |  |  |
| 3–<12 mo | 6016 (100) | 28,146 (100) | 332 (5.5) | 244 (0.9) | 6.4 (5.7–7.0) | 1.0 (0.8–1.1) | 7.06 (5.94–8.38) | 4.18 (3.37–5.17) |
| 1–<5y | 5647 (93.9) | 27,859 (99.0) | 695 (12.3) | 1408 (5.1) | 35.3 (32.7–37.9) | 13.7 (13.0–14.4) | 2.89 (2.62–3.18) | 2.18 (1.94–2.45) |
| 5–<10 y | 4220 (70.1) | 23,292 (82.8) | 562 (13.3) | 1795 (7.7) | 32.0 (29.3–34.6) | 17.5 (16.7–18.3) | 2.34 (2.10–2.60) | 1.83 (1.62–2.06) |
| ≥10 y | 2894 (48.1) | 17,976 (63.9) | 1363 (47.1) | 7026 (39.1) | 40.4 (38.3–42.6) | 28.6 (27.9–29.2) | 2.20 (2.05–2.36) | 1.96 (1.81–2.11) |
| ≥1 y | 5647 (93.9) | 27,859 (99.0) | 2620 (46.4) | 10,229 (36.7) | 36.9 (35.5–38.3) | 22.7 (22.2–23.1) | 2.40 (2.28–2.53) | 1.98 (1.87–2.09) |
| Sex |  |  |  |  |  |  |  |  |
| Female | 3644 (60.6) | 17,134 (60.9) | 1704 (46.8) | 6012 (35.1) | 38.7 (36.8–40.5) | 21.9 (21.3–22.4) | 2.62 (2.46–2.80) | 2.14 (1.99–2.29) |
| Male | 2372 (39.4) | 11,012 (39.1) | 1248 (52.6) | 4461 (40.5) | 38.1 (36.0–40.2) | 21.8 (21.2–22.5) | 2.62 (2.43–2.82) | 2.01 (1.84–2.18) |
| Age at diagnosis, restricting follow-up to 5 y |  |  |  |  |  |  |  |  |
| 18–<30 y | 615 (10.2) | 2825 (10.0) | 14 (2.3) | 12 (0.4) | 4.9 (2.4–7.5) | 0.9 (0.4–1.4) | 5.25 (2.42–11.40) | 10.67 (2.89–39.37) |
| 30–<40 y | 775 (12.9) | 3573 (12.7) | 35 (4.5) | 19 (0.5) | 9.8 (6.6–13.0) | 1.1 (0.6–1.6) | 9.70 (5.36–17.55) | 3.98 (1.77–8.99) |
| 40–<50 y | 992 (16.5) | 4736 (16.8) | 67 (6.8) | 53 (1.1) | 14.8 (11.3–18.4) | 2.3 (1.7–3.0) | 6.46 (4.44–9.40) | 6.56 (3.37–12.76) |
| 50–<60 y | 1254 (20.8) | 5991 (21.3) | 151 (12.0) | 178 (3.0) | 27.3 (22.9–31.6) | 6.3 (5.4–7.2) | 4.40 (3.52–5.51) | 2.98 (2.09–4.25) |
| 60–<70 y | 1354 (22.5) | 6422 (22.8) | 305 (22.5) | 458 (7.1) | 55.3 (49.1–61.5) | 15.5 (14.1–16.9) | 3.76 (3.22–4.38) | 2.41 (1.95–2.97) |
| ≥70 y | 1026 (17.1) | 4599 (16.3) | 455 (44.3) | 932 (20.3) | 127.3 (115.6–139.0) | 47.2 (44.2–50.3) | 2.95 (2.61–3.34) | 2.31 (2.02–2.64) |
| Year of diagnosis |  |  |  |  |  |  |  |  |
| 1969–1986 | 2019 (33.6) | 9802 (34.8) | 1548 (76.7) | 6070 (61.9) | 45.3 (43.0–47.5) | 26.1 (25.5–26.8) | 2.58 (2.42–2.76) | 1.96 (1.81–2.11) |
| 1987–2001 | 1974 (32.8) | 9307 (33.1) | 1099 (55.7) | 3705 (39.8) | 36.3 (34.1–38.4) | 19.9 (19.3–20.6) | 2.69 (2.49–2.91) | 2.14 (1.96–2.33) |
| 2002–2017 | 2023 (33.6) | 9037 (32.1) | 305 (15.1) | 698 (7.7) | 24.7 (22.0–27.5) | 11.4 (10.6–12.3) | 2.57 (2.22–2.97) | 2.29 (1.95–2.68) |
| 1997–2017 | 2248 (37.4) | 10,063 (35.8) | 403 (17.9) | 980 (9.7) | 26.6 (24.0–29.2) | 12.6 (11.8–13.4) | 2.67 (2.34–3.03) | 2.35 (2.04–2.70) |
| Year of diagnosis, restricting follow-up to 5 y |  |  |  |  |  |  |  |  |
| 1969–1986 | 2019 (33.6) | 9802 (34.8) | 438 (21.7) | 721 (7.4) | 49.7 (45.1–54.4) | 15.3 (14.2–16.4) | 3.64 (3.21–4.13) | 2.01 (1.67–2.41) |
| 1987–2001 | 1974 (32.8) | 9307 (33.1) | 368 (18.6) | 556 (6.0) | 42.5 (38.1–46.8) | 12.3 (11.3–13.4) | 3.92 (3.40–4.52) | 2.76 (2.33–3.27) |
| 2002–2012 | 1333 (22.2) | 5957 (21.2) | 179 (13.4) | 301 (5.1) | 29.8 (25.5–34.2) | 10.4 (9.2–11.6) | 3.24 (2.66–3.96) | 2.92 (2.35–3.62) |
| Country of birth |  |  |  |  |  |  |  |  |
| Nordic | 5673 (94.3) | 26,411 (93.8) | 2815 (49.6) | 10,110 (38.3) | 38.7 (37.2–40.1) | 22.2 (21.8–22.6) | 2.61 (2.48–2.74) | 2.06 (1.95–2.18) |
| Other | 341 (5.7) | 1733 (6.2) | 136 (39.9) | 363 (20.9) | 33.8 (28.1–39.4) | 15.0 (13.5–16.5) | 4.20 (1.98–8.88) | 1.77 (0.67–4.63) |
| Education |  |  |  |  |  |  |  |  |
| ≤9 y | 1937 (32.2) | 9785 (34.8) | 1261 (65.1) | 5177 (52.9) | 46.0 (43.5–48.6) | 28.4 (27.6–29.2) | 2.45 (2.24–2.68) | 2.02 (1.84–2.22) |
| 10–12 y | 2171 (36.1) | 10,213 (36.3) | 718 (33.1) | 2380 (23.3) | 22.9 (21.2–24.6) | 13.3 (12.8–13.8) | 2.71 (2.36–3.11) | 2.37 (2.05–2.74) |
| >12 y | 1177 (19.6) | 6022 (21.4) | 260 (22.1) | 859 (14.3) | 17.7 (15.5–19.8) | 8.5 (7.9–9.1) | 3.03 (2.25–4.07) | 2.37 (1.71–3.28) |
| Missing | 731 (12.2) | 2126 (7.6) | 713 (97.5) | 2057 (96.8) | 212.3 (196.7–227.9) | 121.7 (116.4–126.9) | 1.94 (1.69–2.22) | 1.57 (1.35–1.82) |
| Pathology findings |  |  |  |  |  |  |  |  |
| Cirrhosis (stage F4) | 823 (13.7) | 3847 (13.7) | 521 (63.3) | 1736 (45.1) | 69.8 (63.8–75.7) | 26.5 (25.3–27.8) | 4.98 (4.37–5.67) | 3.81 (3.29–4.41) |
| Fibrosis (stage F1–F3) | 1278 (21.2) | 5803 (20.6) | 318 (24.9) | 950 (16.4) | 29.1 (25.9–32.3) | 15.3 (14.3–16.2) | 2.62 (2.27–3.04) | 2.31 (1.96–2.71) |
| Inflammation without fibrosis (stage F0) | 2068 (34.4) | 9720 (34.5) | 1022 (49.4) | 3689 (38.0) | 35.7 (33.5–37.9) | 21.3 (20.6–22.0) | 2.47 (2.28–2.68) | 1.99 (1.82–2.17) |
| Other or unspecified pathology findings | 1847 (30.7) | 8776 (31.2) | 1091 (59.1) | 4098 (46.7) | 36.6 (34.4–38.7) | 23.0 (22.3–23.7) | 2.20 (2.04–2.38) | 1.71 (1.56–1.87) |
| Necrosis | 148 (2.5) | 684 (2.4) | 63 (42.6) | 209 (30.6) | 30.2 (22.7–37.7) | 17.8 (15.4–20.2) | 1.92 (1.40–2.63) | 1.92 (1.33–2.77) |
| Severity of liver disease |  |  |  |  |  |  |  |  |
| Portal hypertension | 294 (4.9) | 1363 (4.8) | 183 (62.2) | 554 (40.6) | 105.2 (90.0–120.4) | 27.5 (25.2–29.8) | 7.79 (6.08–9.99) | 5.83 (4.36–7.81) |
| Liver failure | 157 (2.6) | 744 (2.6) | 74 (47.1) | 254 (34.1) | 36.2 (27.9–44.4) | 18.5 (16.3–20.8) | 3.28 (2.39–4.51) | 2.35 (1.63–3.41) |
| Overlap syndromes |  |  |  |  |  |  |  |  |
| PSC | 92 (1.5) | 415 (1.5) | 17 (18.5) | 31 (7.5) | 30.7 (16.1–45.3) | 6.4 (4.1–8.6) | 8.54 (3.67–19.89) | 57.74 (2.20–1514.16) |
| PBC | 247 (4.1) | 1121 (4.0) | 85 (34.4) | 219 (19.5) | 52.2 (41.1–63.3) | 18.2 (15.8–20.7) | 4.98 (3.61–6.86) | 4.61 (3.25–6.53) |
| Comorbidities^[c](https://www.sciencedirect.com/science/article/pii/S1542356520313951?via%3Dihub#tblS5fnc)^ |  |  |  |  |  |  |  |  |
| CVD | 829 (13.8) | 1710 (6.1) | 590 (71.2) | 1036 (60.6) | 102.5 (94.3–110.8) | 71.8 (67.5–76.2) | 1.60 (1.28–2.01) | 1.48 (1.16–1.89) |
| Malignancy | 422 (7.0) | 822 (2.9) | 296 (70.1) | 397 (48.3) | 107.8 (95.5–120.1) | 57.5 (51.9–63.2) | 2.14 (1.14–4.02) | 2.08 (1.00–4.33) |
| Diabetes | 612 (10.2) | 594 (2.1) | 423 (69.1) | 320 (53.9) | 86.3 (78.1–94.6) | 73.5 (65.4–81.5) | 1.47 (0.90–2.39) | 1.69 (0.92–3.11) |
| ESRD | 13 (0.2) | 24 (0.1) | 11 (84.6) | 10 (41.7) | 105.5 (43.1–167.8) | 74.6 (28.4–120.9) | — | — |
| Other autoimmune diseases | 874 (14.5) | 474 (1.7) | 265 (30.3) | 143 (30.2) | 33.5 (29.5–37.5) | 37.5 (31.4–43.6) | 1.95 (0.92–4.13) | 2.20 (0.87–5.60) |
| Presence of conditions that affect use of first-line medical treatment | 488 (8.1) | 801 (2.8) | 243 (49.8) | 180 (22.5) | 44.7 (39.1–50.4) | 14.6 (12.5–16.8) | 2.86 (1.12–7.35) | 3.64 (0.75–17.56) |

NOTE. Values are n (%), unless otherwise indicated.

AIH, autoimmune hepatitis; CI, confidence interval; CVD, cardiovascular disease; [ESRD](https://www.sciencedirect.com/topics/medicine-and-dentistry/end-stage-renal-disease), end-stage renal disease; HR, hazard ratio; IQR, interquartile range; [PBC](https://www.sciencedirect.com/topics/medicine-and-dentistry/primary-biliary-cirrhosis), primary biliary cholangitis; [PSC](https://www.sciencedirect.com/topics/medicine-and-dentistry/primary-sclerosing-cholangitis), primary sclerosing cholangitis; PY, person-years.

a

Conditioned on age, sex, county, and calendar period.

b

Conditioned and further adjusted for education and baseline medical comorbidities (CVD, non-liver cancer, diabetes, end stage renal disease, and other autoimmune disease) and factors that would preclude treatment with first-line therapy (infection, psychosis, pregnancy, tuberculosis, lymphoma, and vertebral compression fracture).

c

CVD, malignancy, diabetes, ESRD, tuberculosis, lymphoma, vertebral [compression fracture](https://www.sciencedirect.com/topics/medicine-and-dentistry/compression-fracture), and other autoimmune disease status within 5 years before index date; infection, psychosis, and pregnancy status within 1 year before index date.

Supplementary Table 6. Characteristics of Excluded Patients With AIH With <3 Months of Follow-Up and Their Matched General Population Comparators

| **Group** | **Cases** | **Controls** |
| --- | --- | --- |
| N | 422 | 1997 |
| Follow-up |  |  |
| Mean (SD) | 0.1 (0.1) | 13.3 (9.0) |
| Median (IQR) | 0.1 (0.0–0.1) | 11.8 (5.9–19.7) |
| Range (minimum–maximum) | 0.0–0.2 | 0.0–38.7 |
| Sex |  |  |
| Female | 199 (47.2) | 947 (47.4) |
| Male | 223 (52.8) | 1050 (52.6) |
| Age, y |  |  |
| Mean (SD) | 67.4 (12.4) | 67.3 (12.3) |
| Median (IQR) | 69.6 (61.1–75.9) | 69.5 (61.0–75.6) |
| Range (minimum–maximum) | 19.1–89.1 | 18.5–89.2 |
| Age category |  |  |
| 18–<30 y | 4 (0.9) | 19 (1.0) |
| 30–<40 y | 14 (3.3) | 59 (3.0) |
| 40–<50 y | 21 (5.0) | 98 (4.9) |
| 50–<60 y | 58 (13.7) | 281 (14.1) |
| 60–<70 y | 124 (29.4) | 583 (29.2) |
| ≥70 y | 201 (47.6) | 957 (47.9) |
| Year of diagnosis^[a](https://www.sciencedirect.com/science/article/pii/S1542356520313951?via%3Dihub#tblS6fn)^ |  |  |
| 1969–1986 | 152 (36.0) | 735 (36.8) |
| 1987–2001 | 176 (41.7) | 843 (42.2) |
| 2002–2017 | 94 (22.3) | 419 (21.0) |
| Time to register-based definition of AIH onset (time in years between first AIH diagnosis and biopsy) |  |  |
| Mean (SD) | 6.9 (9.1) |  |
| Median (IQR) | 2.1 (0.1–11.7) |  |
| Range (minimum–maximum) | 0.0–40.2 |  |
| Country of birth |  |  |
| Nordic | 401 (95.0) | 1902 (95.2) |
| Other | 21 (5.0) | 95 (4.8) |
| Level of education |  |  |
| ≤9 y | 109 (25.8) | 855 (42.8) |
| 10–12 y | 75 (17.8) | 492 (24.6) |
| >12 y | 24 (5.7) | 202 (10.1) |
| Missing | 214 (50.7) | 448 (22.4) |
| Level of education using highest level of education in parents when missing |  |  |
| ≤9 y | 115 (27.3) | 856 (42.9) |
| 10–12 y | 77 (18.2) | 492 (24.6) |
| >12 y | 24 (5.7) | 202 (10.1) |
| Missing | 206 (48.8) | 447 (22.4) |
| Pathology findings^[b](https://www.sciencedirect.com/science/article/pii/S1542356520313951?via%3Dihub#tblS6fnb)^ |  |  |
| Cirrhosis (stage F4) | 91 (21.6) |  |
| Fibrosis (stage F1–F3) | 23 (5.5) |  |
| Inflammation without fibrosis (stage F0) | 63 (14.9) |  |
| Other or unspecified pathology findings | 245 (58.1) |  |
| Necrosis | 8 (1.9) |  |
| Severity of liver disease |  |  |
| Portal hypertension | 86 (20.4) |  |
| Liver failure | 20 (4.7) |  |
| AIH treatment during follow-up^[c](https://www.sciencedirect.com/science/article/pii/S1542356520313951?via%3Dihub#tblS6fnc)^ |  |  |
| Second-line medications | 0 |  |
| Azathioprine | 1 (1.5) |  |
| Prednisolone/prednisone | 5 (7.8) |  |
| Overlap syndromes |  |  |
| PSC | 7 (1.7) |  |
| PBC | 19 (4.5) |  |
| Comorbidities^[d](https://www.sciencedirect.com/science/article/pii/S1542356520313951?via%3Dihub#tblS6fnd)^ |  |  |
| CVD | 136 (32.2) | 255 (12.8) |
| Extrahepatic malignancy | 178 (42.2) | 94 (4.7) |
| Diabetes | 85 (20.1) | 82 (4.1) |
| ESRD | 3 (0.7) | 2 (0.1) |
| Other autoimmune disease | 50 (11.8) | 41 (2.1) |
| ^[e](https://www.sciencedirect.com/science/article/pii/S1542356520313951?via%3Dihub#tblS6fne)^Presence of conditions that affect use of first-line medical treatment | 63 (14.9) | 36 (1.8) |

NOTE. Values are n (%), unless otherwise indicated.

AIH, autoimmune hepatitis; CVD, cardiovascular disease; [ESRD](https://www.sciencedirect.com/topics/medicine-and-dentistry/end-stage-renal-disease), end-stage renal disease; IQR, interquartile range; [PBC](https://www.sciencedirect.com/topics/medicine-and-dentistry/primary-biliary-cirrhosis), primary biliary cholangitis; [PSC](https://www.sciencedirect.com/topics/medicine-and-dentistry/primary-sclerosing-cholangitis), primary sclerosing cholangitis; PY, person-years.

a

Last of [liver biopsy](https://www.sciencedirect.com/topics/medicine-and-dentistry/liver-biopsy) or relevant AIH [International Classification of Diseases](https://www.sciencedirect.com/topics/medicine-and-dentistry/international-classification-of-diseases) code.

b

[Cirrhosis](https://www.sciencedirect.com/topics/medicine-and-dentistry/liver-cirrhosis), fibrosis, inflammation without fibrosis, and other are mutually exclusive, while necrosis is not mutually exclusive.

c

Restricted to individuals with an incident AIH diagnosis on January 1, 2006, or later (AIH = 1744).

d

CVD, malignancy, diabetes, ESRD, tuberculosis, lymphoma, vertebral [compression fracture](https://www.sciencedirect.com/topics/medicine-and-dentistry/compression-fracture), and other autoimmune disease status within 5 years before index date; infection, psychosis, and pregnancy status within 1 year before index date.

e

Factors that would preclude treatment with first-line therapy (infection, psychosis, pregnancy, tuberculosis, lymphoma, and vertebral compression fracture).

Supplementary Table 7. Risk of All-Cause Mortality in Patients With AIH and Matched General Population Comparators Including First 3 Months of Follow-Up

| **Group** | **Patients** | | **Events** | | **Incidence rate (95% CI) per 1000 PY** | | **HR (95% CI)****^[a](https://www.sciencedirect.com/science/article/pii/S1542356520313951?via%3Dihub#tblS7fna)^** | **HR (95% CI)****^[b](https://www.sciencedirect.com/science/article/pii/S1542356520313951?via%3Dihub#tblS7fnb)^** |
| --- | --- | --- | --- | --- | --- | --- | --- | --- |
|  | **AIH** | **Comparators** | **AIH** | **Comparators** | **AIH** | **Comparators** |  |  |
| Follow-up |  |  |  |  |  |  |  |  |
| ≥0 mo | 6438 (100) | 30,233 (100) | 3605 (56.0) | 11,899 (39.4) | 46.9 (45.4–48.4) | 23.5 (23.1–23.9) | 3.23 (3.09–3.38) | 2.52 (2.39–2.65) |
| 0–<3 mo | 6438 (100) | 30,233 (100) | 420 (6.5) | 94 (0.3) | 22.7 (20.5–24.8) | 1.0 (0.8–1.2) | 23.81 (18.81–30.14) | 14.97 (11.29–19.85) |
| ≥3 mo | 6016 (100) | 28,146 (100) | 3185 (52.9) | 10,477 (37.2) | 41.4 (40.0–42.9) | 21.9 (21.4–22.3) | 2.86 (2.72–2.99) | 2.29 (2.17–2.41) |

NOTE. Values are n (%), unless otherwise indicated.

AIH, autoimmune hepatitis; CI, confidence interval; HR, hazard ratio; PY, person-years.

a

Conditioned on age, sex, county, and calendar period.

b

Conditioned and further adjusted for education and baseline medical comorbidities (CVD, non-liver cancer, diabetes, [end stage renal disease](https://www.sciencedirect.com/topics/medicine-and-dentistry/end-stage-renal-disease), and other autoimmune disease) and factors that would preclude treatment with first-line therapy (infection, psychosis, pregnancy, lymphoma, and vertebral compression fracture).

Supplementary Table 8. Risk of All-Cause And Cancer-Specific Mortality in Patients With AIH and Matched General Population Comparators With No History of Cancer Before Index Date

|  | **All-cause mortality** | | **Cancer** | |
| --- | --- | --- | --- | --- |
|  | **AIH** | **Comparators** | **AIH** | **Comparators** |
| N | 5433 | 24,404 | 5401 | 24,270 |
| Death or liver transplantation, | 2786 (51.3) | 8774 (36.0) | 557 (10.3) | 2095 (8.6) |
| Death | 2566 (47.2) | 8771 (35.9) | 557 (10.3) | 2095 (8.6) |
| Liver transplantation | 220 (4.0) | 3 (0.0) | 0 (0.0) | 0 (0.0) |
| Follow-up, y |  |  |  |  |
| Mean (SD) | 13.5 (10.3) | 17.8 (11.0) | 13.1 (10.2) | 17.3 (10.9) |
| Median (IQR) | 10.3 (4.7–21.7) | 17.3 (7.6–27.3) | 9.9 (4.2–21.3) | 17.3 (7.0–26.8) |
| Range (minimum–maximum) | 0.3–43.1 | 0.3–47.8 | 0.3–42.8 | 0.3–46.8 |
| Incidence rate per 1000 PY (95% CI) | 38.1 (36.7–39.5) | 20.2 (19.7–20.6) | 7.9 (7.2–8.6) | 5.0 (4.8–5.2) |
| HR (95% CI) |  |  |  |  |
| Conditioned^[a](https://www.sciencedirect.com/science/article/pii/S1542356520313951?via%3Dihub#tblS8fna)^ | 2.79 (2.65–2.94) | | 2.06 (1.86–2.29) | |
| Adjusted^[b](https://www.sciencedirect.com/science/article/pii/S1542356520313951?via%3Dihub#tblS8fnb)^ | 2.26 (2.14–2.39) | | 1.89 (1.69–2.13) | |

NOTE. Values are n (%), unless otherwise indicated.

AIH, autoimmune hepatitis; CI, confidence interval; HR, hazard ratio; IQR, interquartile range; PY, person-years.

a

Conditioned on age, sex, county, and calendar period.

b

Conditioned and further adjusted for education and baseline medical comorbidities (CVD, non-liver cancer, diabetes, [end stage renal disease](https://www.sciencedirect.com/topics/medicine-and-dentistry/end-stage-renal-disease), and other autoimmune disease) and factors that would preclude treatment with first-line therapy (infection, psychosis, pregnancy, tuberculosis, lymphoma, and vertebral compression fracture).

Supplementary Table 9. Median Time of Transplant-Free All-Cause Mortality (Follow-Up Until December 31, 2017) by Age in Patients With AIH and in Matched General Population Comparators

| **Group** | **Patients** | | **Median transplant-free all-cause Mortality survival time (95% CI) (y)** | |
| --- | --- | --- | --- | --- |
|  | **AIH** | **Comparators** |  |  |
| Overall (≥3 mo) | 6016 (100) | 28,146 (100) | 17.6 (17.0–18.2) | 29.1 (28.7–29.6) |
| Age |  |  |  |  |
| 18–<30 y | 615 (10.2) | 2825 (10.0) | 38.1 (–) | — |
| 30–<40 y | 775 (12.9) | 3573 (12.7) | 33.4 (32.1–36.6) | — |
| 40–<50 y | 992 (16.5) | 4736 (16.8) | 26.3 (25.0–28.6) | 39.4 (38.1–40.4) |
| 50–<60 y | 1254 (20.8) | 5991 (21.3) | 19.0 (17.7–20.5) | 29.7 (29.2–30.1) |
| 60–<70 y | 1354 (22.5) | 6422 (22.8) | 12.6 (11.5–13.5) | 19.2 (18.9–19.5) |
| ≥70 y | 1026 (17.1) | 4599 (16.3) | 5.6 (5.0–6.0) | 10.5 (10.3–10.9) |

NOTE. Values are n (%), unless otherwise indicated.

AIH, autoimmune hepatitis; CI, confidence interval.

Supplementary Table 10. Risk of All-Cause Mortality in Patients With AIH and Matched General Population Comparators: Restricted to AIH Patients With Time Between Biopsy to Diagnosis <1 Year

| **Group** | **Patients** | | **Events** | | **Incidence rate (95% CI) per 1000 PY** | | **HR (95% CI)****^[a](https://www.sciencedirect.com/science/article/pii/S1542356520313951?via%3Dihub#tblS10fna)^** | **HR (95% CI)****^[b](https://www.sciencedirect.com/science/article/pii/S1542356520313951?via%3Dihub#tblS10fnb)^** |
| --- | --- | --- | --- | --- | --- | --- | --- | --- |
|  | **AIH** | **Comparators** | **AIH** | **Comparators** | **AIH** | **Comparators** |  |  |
| Overall (≥3 mo) | 3934 (100) | 18,682 (100) | 2103 (53.5) | 7489 (40.1) | 38.0 (36.4–39.6) | 22.4 (21.9–22.9) | 2.41 (2.28–2.56) | 2.00 (1.88–2.13) |
| Follow-up |  |  |  |  |  |  |  |  |
| 3–<12 mo | 3934 (100) | 18,682 (100) | 187 (4.8) | 165 (0.9) | 5.4 (4.7–6.2) | 1.0 (0.8–1.1) | 5.95 (4.80–7.38) | 3.73 (2.87–4.85) |
| 1–<5 y | 3743 (95.1) | 18,489 (99.0) | 425 (11.4) | 965 (5.2) | 31.9 (28.9–35.0) | 14.1 (13.2–15.0) | 2.51 (2.22–2.82) | 1.92 (1.66–2.23) |
| 5–<10 y | 2891 (73.5) | 15,498 (83.0) | 395 (13.7) | 1225 (7.9) | 32.1 (29.0–35.3) | 17.8 (16.8–18.8) | 2.25 (1.98–2.55) | 1.87 (1.61–2.16) |
| ≥10 y | 2087 (53.1) | 12,294 (65.8) | 1096 (52.5) | 5134 (41.8) | 42.3 (39.8–44.8) | 28.7 (28.0–29.5) | 2.16 (2.00–2.35) | 1.96 (1.80–2.13) |
| ≥1 y | 3743 (95.1) | 18,489 (99.0) | 1916 (51.2) | 7324 (39.6) | 37.2 (35.6–38.9) | 23.2 (22.7–23.7) | 2.26 (2.13–2.40) | 1.93 (1.81–2.06) |
| Sex |  |  |  |  |  |  |  |  |
| Female | 2317 (58.9) | 11,046 (59.1) | 1163 (50.2) | 4133 (37.4) | 38.3 (36.1–40.5) | 22.5 (21.8–23.2) | 2.43 (2.25–2.62) | 2.07 (1.91–2.25) |
| Male | 1617 (41.1) | 7636 (40.9) | 940 (58.1) | 3356 (43.9) | 37.7 (35.3–40.1) | 22.3 (21.5–23.0) | 2.39 (2.20–2.61) | 1.90 (1.73–2.10) |
| Age at diagnosis, restricting follow-up to 5 y |  |  |  |  |  |  |  |  |
| 18–<30 y | 401 (10.2) | 1824 (9.8) | 13 (3.2) | 9 (0.5) | 6.9 (3.2–10.7) | 1.0 (0.4–1.7) | 6.52 (2.78–15.28) | 10.85 (2.82–41.73) |
| 30–<40 y | 542 (13.8) | 2516 (13.5) | 33 (6.1) | 17 (0.7) | 13.0 (8.6–17.5) | 1.4 (0.7–2.1) | 10.20 (5.45–19.09) | 6.61 (2.87–15.23) |
| 40–<50 y | 646 (16.4) | 3118 (16.7) | 41 (6.3) | 37 (1.2) | 13.6 (9.4–17.8) | 2.5 (1.7–3.3) | 5.54 (3.50–8.77) | 6.39 (2.97–13.78) |
| 50–<60 y | 862 (21.9) | 4150 (22.2) | 98 (11.4) | 134 (3.2) | 25.2 (20.2–30.2) | 6.8 (5.7–8.0) | 3.70 (2.84–4.83) | 2.26 (1.45–3.51) |
| 60–<70 y | 887 (22.5) | 4239 (22.7) | 179 (20.2) | 311 (7.3) | 48.2 (41.2–55.3) | 16.0 (14.2–17.7) | 3.09 (2.55–3.74) | 1.97 (1.49–2.59) |
| ≥70 y | 596 (15.1) | 2835 (15.2) | 248 (41.6) | 622 (21.9) | 116.5 (102.0–131.0) | 51.5 (47.5–55.6) | 2.42 (2.07–2.83) | 1.99 (1.67–2.36) |
| Year of diagnosis |  |  |  |  |  |  |  |  |
| 1969–1986 | 1642 (41.7) | 8002 (42.8) | 1294 (78.8) | 4934 (61.7) | 44.3 (41.9–46.7) | 25.9 (25.2–26.6) | 2.45 (2.27–2.63) | 1.99 (1.83–2.16) |
| 1987–2001 | 1167 (29.7) | 5558 (29.8) | 667 (57.2) | 2233 (40.2) | 34.6 (32.0–37.2) | 20.1 (19.3–21.0) | 2.38 (2.15–2.63) | 1.99 (1.79–2.21) |
| 2002–2017 | 1125 (28.6) | 5122 (27.4) | 142 (12.6) | 322 (6.3) | 20.8 (17.4–24.2) | 9.8 (8.7–10.8) | 2.32 (1.88–2.86) | 2.06 (1.64–2.58) |
| 1997–2017 | 1191 (30.3) | 5434 (29.1) | 164 (13.8) | 397 (7.3) | 21.0 (17.8–24.2) | 10.5 (9.4–11.5) | 2.23 (1.84–2.71) | 1.97 (1.60–2.43) |
| Year of diagnosis, restricting follow-up to 5 y |  |  |  |  |  |  |  |  |
| 1969–1986 | 1642 (41.7) | 8002 (42.8) | 312 (19.0) | 591 (7.4) | 42.7 (38.0–47.4) | 15.4 (14.1–16.6) | 3.14 (2.72–3.64) | 1.93 (1.57–2.38) |
| 1987–2001 | 1167 (29.7) | 5558 (29.8) | 200 (17.1) | 354 (6.4) | 37.9 (32.7–43.2) | 13.2 (11.8–14.5) | 3.14 (2.61–3.76) | 2.38 (1.92–2.93) |
| 2002–2012 | 694 (17.6) | 3154 (16.9) | 78 (11.2) | 141 (4.5) | 24.2 (18.8–29.6) | 9.2 (7.6–10.7) | 2.89 (2.17–3.86) | 2.59 (1.87–3.57) |
| Country of birth |  |  |  |  |  |  |  |  |
| Nordic | 3687 (93.7) | 17,536 (93.9) | 1993 (54.1) | 7231 (41.2) | 38.2 (36.5–39.9) | 22.8 (22.2–23.3) | 2.39 (2.25–2.54) | 1.98 (1.85–2.11) |
| Other | 245 (6.2) | 1144 (6.1) | 109 (44.5) | 258 (22.6) | 34.2 (27.8–40.7) | 15.3 (13.4–17.2) | 3.43 (1.52–7.72) | 1.81 (0.62–5.25) |
| Education |  |  |  |  |  |  |  |  |
| ≤9 y | 1305 (33.2) | 6603 (35.3) | 880 (67.4) | 3632 (55.0) | 42.6 (39.8–45.4) | 27.6 (26.7–28.5) | 2.18 (1.97–2.43) | 1.85 (1.65–2.06) |
| 10–12 y | 1370 (34.8) | 6578 (35.2) | 524 (38.2) | 1640 (24.9) | 23.8 (21.7–25.8) | 13.4 (12.8–14.1) | 2.66 (2.26–3.12) | 2.44 (2.06–2.89) |
| >12 y | 732 (18.6) | 3818 (20.4) | 183 (25.0) | 583 (15.3) | 18.4 (15.7–21.0) | 8.6 (7.9–9.3) | 2.52 (1.79–3.56) | 2.04 (1.40–2.97) |
| Missing | 527 (13.4) | 1683 (9.0) | 516 (97.9) | 1634 (97.1) | 194.1 (177.4–210.9) | 121.5 (115.7–127.4) | 1.78 (1.53–2.09) | 1.49 (1.26–1.76) |
| Pathology findings |  |  |  |  |  |  |  |  |
| Cirrhosis (stage F4) | 484 (12.3) | 2309 (12.4) | 364 (75.2) | 1126 (48.8) | 80.6 (72.3–88.9) | 28.2 (26.6–29.9) | 5.27 (4.50–6.17) | 4.09 (3.43–4.87) |
| Fibrosis (stage F1–F3) | 810 (20.6) | 3729 (20.0) | 226 (27.9) | 645 (17.3) | 31.9 (27.7–36.1) | 16.0 (14.8–17.3) | 2.77 (2.33–3.31) | 2.45 (2.02–2.98) |
| Inflammation without fibrosis (stage F0) | 1359 (34.5) | 6485 (34.7) | 744 (54.7) | 2692 (41.5) | 35.9 (33.3–38.5) | 22.0 (21.2–22.8) | 2.34 (2.13–2.57) | 1.96 (1.76–2.17) |
| Other or unspecified pathology findings | 1281 (32.6) | 6159 (33.0) | 769 (60.0) | 3026 (49.1) | 33.4 (31.0–35.8) | 22.9 (22.1–23.8) | 1.86 (1.70–2.04) | 1.55 (1.40–1.71) |
| Necrosis | 112 (2.8) | 528 (2.8) | 50 (44.6) | 172 (32.6) | 28.4 (20.6–36.3) | 17.7 (15.0–20.3) | 1.85 (1.30–2.63) | 1.76 (1.16–2.68) |
| Severity of liver disease |  |  |  |  |  |  |  |  |
| Portal hypertension | 150 (3.8) | 710 (3.8) | 107 (71.3) | 318 (44.8) | 106.7 (86.5–126.9) | 30.2 (26.8–33.5) | 6.44 (4.75–8.75) | 5.26 (3.67–7.55) |
| Liver failure | 79 (2.0) | 381 (2.0) | 39 (49.4) | 131 (34.4) | 37.8 (25.9–49.7) | 19.2 (15.9–22.5) | 3.13 (2.03–4.82) | 2.39 (1.43–4.00) |
| Overlap syndromes |  |  |  |  |  |  |  |  |
| PSC | 24 (0.6) | 109 (0.6) | 6 (25.0) | (0.0) | 40.4 (8.1–72.8) | 0.0 (0.0–0.0) | - | - |
| PBC | 76 (1.9) | 351 (1.9) | 17 (22.4) | 41 (11.7) | 41.3 (21.7–60.9) | 17.1 (11.9–22.4) | 4.28 (2.10–8.73) | 3.76 (1.75–8.07) |
| Comorbidities^[c](https://www.sciencedirect.com/science/article/pii/S1542356520313951?via%3Dihub#tblS10fnc)^ |  |  |  |  |  |  |  |  |
| CVD | 516 (13.1) | 1055 (5.6) | 374 (72.5) | 677 (64.2) | 94.7 (85.1–104.4) | 75.2 (69.5–80.8) | 1.26 (0.96–1.67) | 1.17 (0.86–1.59) |
| Malignancy | 223 (5.7) | 515 (2.8) | 155 (69.5) | 264 (51.3) | 94.7 (79.8–109.6) | 59.5 (52.4–66.7) | 2.17 (0.97–4.86) | 2.16 (0.83–5.61) |
| Diabetes | 361 (9.2) | 367 (2.0) | 265 (73.4) | 214 (58.3) | 83.3 (73.3–93.4) | 76.7 (66.4–87.0) | 1.58 (0.84–2.99) | 2.11 (0.92–4.82) |
| ESRD | 4 (0.1) | 12 (0.1) | 4 (100.0) | 5 (41.7) | 100.5 (2.0–199.0) | 79.1 (9.8–148.4) | - | - |
| Other autoimmune diseases | 432 (11.0) | 258 (1.4) | 145 (33.6) | 85 (32.9) | 33.8 (28.3–39.3) | 38.4 (30.2–46.6) | 1.00 (0.33–3.01) | 0.17 (0.01–2.95) |
| Presence of conditions that affect use of first-line medical treatment | 307 (7.8) | 494 (2.6) | 168 (54.7) | 117 (23.7) | 42.6 (36.2–49.0) | 14.6 (11.9–17.2) | 2.64 (0.82–8.46) | 4.10 (0.71–23.68) |

NOTE. Values are n (%), unless otherwise indicated.

AIH, autoimmune hepatitis; CI, confidence interval; CVD, cardiovascular disease; [ESRD](https://www.sciencedirect.com/topics/medicine-and-dentistry/end-stage-renal-disease), end-stage renal disease; HR, hazard ratio; [PBC](https://www.sciencedirect.com/topics/medicine-and-dentistry/primary-biliary-cirrhosis), primary biliary cholangitis; [PSC](https://www.sciencedirect.com/topics/medicine-and-dentistry/primary-sclerosing-cholangitis), primary sclerosing cholangitis; PY, person-years.

a

Conditioned on age, sex, county, and calendar period.

b

Conditioned and further adjusted for education and baseline medical comorbidities (CVD, non-liver cancer, diabetes, end stage renal disease, and other autoimmune disease) and factors that would preclude treatment with first-line therapy (infection, psychosis, pregnancy, tuberculosis, lymphoma, and vertebral compression fracture).

c

CVD, malignancy, diabetes, ESRD, tuberculosis, lymphoma, vertbral [compression fracture](https://www.sciencedirect.com/topics/medicine-and-dentistry/compression-fracture), and other autoimmune disease status within 5 years before index date; infection, psychosis, and pregnancy status within 1 year before index date.

Supplementary Table 11. Characteristics of Patients With AIH and Sibling Comparators

| **Group** | **Cases** | **Siblings** |
| --- | --- | --- |
| N | 2505 | 4908 |
| Follow-up |  |  |
| Mean (SD) | 14.2 (10.7) | 17.4 (11.3) |
| Median (IQR) | 10.4 (4.9–23.7) | 16.3 (6.5–27.6) |
| Range (minimum–maximum) | 0.3–40.8 | 0.4–46.8 |
| Follow-up category |  |  |
| 3–<12 mo | 2505 (100) | 4908 (100) |
| 1–<5 y | 2438 (97.3) | 4897 (99.8) |
| 5–<10 y | 1859 (74.2) | 4031 (82.1) |
| ≥10 y | 1299 (51.9) | 3054 (62.2) |
| Sex |  |  |
| Female | 1513 (60.4) | 2377 (48.4) |
| Male | 992 (39.6) | 2531 (51.6) |
| Age, y |  |  |
| Mean (SD) | 45.2 (14.5) | 45.4 (14.4) |
| Median (IQR) | 44.6 (33.9–55.9) | 44.7 (34.5–56.0) |
| Range (minimum–maximum) | 18.1–81.8 | 18.0–84.4 |
| Age category |  |  |
| 18–<30 y | 433 (17.3) | 800 (16.3) |
| 30–<40 y | 548 (21.9) | 1057 (21.5) |
| 40–<50 y | 584 (23.3) | 1242 (25.3) |
| 50–<60 y | 479 (19.1) | 918 (18.7) |
| 60–<70 y | 347 (13.9) | 638 (13.0) |
| ≥70 y | 114 (4.6) | 253 (5.2) |
| Year of diagnosis^[a](https://www.sciencedirect.com/science/article/pii/S1542356520313951?via%3Dihub#tblS11fna)^ |  |  |
| 1969–1986 | 470 (18.8) | 1005 (20.5) |
| 1987- 2001 | 800 (31.9) | 1648 (33.6) |
| 2002–2017 | 1235 (49.3) | 2255 (45.9) |
| Time to register-based definition of AIH onset (time in years between first AIH diagnosis and biopsy) |  |  |
| Mean (SD) | 3.3 (6.1) |  |
| Median (IQR) | 0.3 (0.0–3.6) |  |
| Range (minimum–maximum) | 0.0–45.5 |  |
| Country of birth |  |  |
| Nordic | 2474 (98.8) | 4833 (98.5) |
| Other | 31 (1.2) | 75 (1.5) |
| Level of education |  |  |
| ≤9 y | 588 (23.5) | 1386 (28.2) |
| 10–12 y | 1188 (47.4) | 2289 (46.6) |
| >12 y | 700 (27.9) | 1211 (24.7) |
| Missing | 29 (1.2) | 22 (0.4) |
| Level of education using highest level of education in parents when missing |  |  |
| ≤9 y | 601 (24.0) | 1391 (28.3) |
| 10–12 y | 1194 (47.7) | 2291 (46.7) |
| >12 y | 707 (28.2) | 1211 (24.7) |
| Missing | 3 (0.1) | 15 (0.3) |
| Pathology findings^[b](https://www.sciencedirect.com/science/article/pii/S1542356520313951?via%3Dihub#tblS11fnb)^ |  |  |
| Cirrhosis (stage F4) | 271 (10.8) |  |
| Fibrosis (stage F1–F3) | 722 (28.8) |  |
| Inflammation without fibrosis (stage F0) | 847 (33.8) |  |
| Other or unspecified pathology findings | 665 (26.5) |  |
| Necrosis | 59 (2.4) |  |
| Severity of liver disease |  |  |
| Portal hypertension | 104 (4.2) |  |
| Liver failure | 66 (2.6) |  |
| AIH treatment during follow-up^[c](https://www.sciencedirect.com/science/article/pii/S1542356520313951?via%3Dihub#tblS11fnc)^ |  |  |
| Second-line medications | 132 (12.1) |  |
| Azathioprine | 429 (39.2) |  |
| Prednisolone/prednisone | 269 (24.5) |  |
| Overlap syndromes |  |  |
| PSC | 63 (2.5) |  |
| PBC | 123 (4.9) |  |
| Comorbidities^[d](https://www.sciencedirect.com/science/article/pii/S1542356520313951?via%3Dihub#tblS11fnd)^ |  |  |
| Cardiovascular disease | 195 (7.8) | 235 (4.8) |
| Malignancy | 126 (5.0) | 138 (2.8) |
| Diabetes | 175 (7.0) | 126 (2.6) |
| End-stage renal disease | 9 (0.4) | 4 (0.1) |
| Other autoimmune disease | 495 (19.8) | 164 (3.3) |
| Presence of conditions that affect use of first-line medical treatment^[e](https://www.sciencedirect.com/science/article/pii/S1542356520313951?via%3Dihub#tblS11fne)^ | 204 (8.1) | 187 (3.8) |

NOTE. Values are n (%), unless otherwise indicated.

AIH, autoimmune hepatitis; IQR, interquartile range; [PBC](https://www.sciencedirect.com/topics/medicine-and-dentistry/primary-biliary-cirrhosis), primary biliary cholangitis; [PSC](https://www.sciencedirect.com/topics/medicine-and-dentistry/primary-sclerosing-cholangitis), primary sclerosing cholangitis.

a

Last of [liver biopsy](https://www.sciencedirect.com/topics/medicine-and-dentistry/liver-biopsy) or relevant AIH [International Classification of Diseases](https://www.sciencedirect.com/topics/medicine-and-dentistry/international-classification-of-diseases) code.

b

[Cirrhosis](https://www.sciencedirect.com/topics/medicine-and-dentistry/liver-cirrhosis), fibrosis, inflammation without fibrosis, and other are mutually exclusive, while necrosis is not mutually exclusive.

c

Restricted to individuals with an incident AIH diagnosis on January 1, 2006, or later (AIH = 1084)

d

Cardiovascular disease, malignancy, diabetes, end-stage renal disease, tuberculosis, lymphoma, vertebral [compression fracture](https://www.sciencedirect.com/topics/medicine-and-dentistry/compression-fracture), and other autoimmune disease status within 5 years before index date; infection, psychosis, and pregnancy status within 1 year before index date.

e

Factors that would preclude treatment with first-line therapy (infection, psychosis, pregnancy, tuberculosis, lymphoma, and vertebral compression fracture).

Supplementary Table 12. Risk of All-Cause and Cause-Specific Mortality in Patients With AIH and Sibling Comparators

|  | **All-cause mortality** | | **CVD** | | **Extrahepatic cancer** | | **Liver related** | | | **Other cause** | |
| --- | --- | --- | --- | --- | --- | --- | --- | --- | --- | --- | --- |
|  | **AIH** | **Siblings** | **AIH** | **Siblings** | **AIH** | **Siblings** | | **AIH** | **Siblings** | **AIH** | **Siblings** |
| N | 2505 | 4908 | 2481 | 4868 | 2481 | 4868 | | 2481 | 4868 | 2481 | 4868 |
| Death or liver transplantation, | 706 (28.2) | 527 (10.7) | 102 (4.1) | 142 (2.9) | 147 (5.9) | 169 (3.5) | | 254 (10.2) | 23 (0.5) | 202 (8.1) | 193 (4.0) |
| Death | 558 (22.3) | 521 (10.6) | 102 (4.1) | 142 (2.9) | 147 (5.9) | 169 (3.5) | | 106 (4.3) | 17 (0.3) | 202 (8.1) | 193 (4.0) |
| Liver transplantation | 148 (5.9) | 6 (0.1) | 0 (0.0) | 0 (0.0) | 0 (0.0) | 0 (0.0) | | 148 (6.0) | 6 (0.1) | 0 (0.0) | 0 (0.0) |
| Follow-up, y |  |  |  |  |  |  | |  |  |  |  |
| Mean (SD) | 14.2 (10.7) | 17.4 (11.3) | 13.6 (10.7) | 16.6 (11.2) | 13.6 (10.7) | 16.6 (11.2) | | 13.6 (10.7) | 16.6 (11.2) | 13.6 (10.7) | 16.6 (11.2) |
| Median (IQR) | 10.4 (4.9–23.7) | 16.3 (6.5–27.6) | 9.9 (4.1–23.2) | 15.6 (5.9–26.7) | 9.9 (4.1–23.2) | 15.6 (5.9–26.7) | | 9.9 (4.1–23.2) | 15.6 (5.9–26.7) | 9.9 (4.1–23.2) | 15.6 (5.9–26.7) |
| Range (minimum–maximum) | 0.3–40.8 | 0.4–46.8 | 0.3–39.8 | 0.3–45.8 | 0.3–39.8 | 0.3–45.8 | | 0.3–39.8 | 0.3–45.8 | 0.3–39.8 | 0.3–45.8 |
| Incidence rate per 1000 PY (95% CI) | 19.9 (18.4–21.4) | 6.2 (5.7–6.7) | 3.0 (2.4–3.6) | 1.8 (1.5–2.0) | 4.4 (3.7–5.1) | 2.1 (1.8–2.4) | | 7.5 (6.6–8.5) | 0.3 (0.2–0.4) | 6.0 (5.2–6.8) | 2.4 (2.0–2.7) |
| HR (95% CI) |  |  |  |  |  |  | |  |  |  |  |
| Conditioned^[a](https://www.sciencedirect.com/science/article/pii/S1542356520313951?via%3Dihub#tblS12fna)^ | 3.73 (3.26–4.26) | | 2.09 (1.56–2.80) | | 2.55 (1.97–3.31) | | 31.05 (18.60–51.84) | | | 2.65 (2.11–3.34) | |
| Adjusted^[b](https://www.sciencedirect.com/science/article/pii/S1542356520313951?via%3Dihub#tblS12fnb)^ | 3.44 (2.97–3.99) | | 1.97 (1.39–2.79) | | 2.17 (1.61–2.93) | | 47.95 (23.05–99.74) | | | 2.40 (1.85–3.11) | |

NOTE. Values are n (%), unless otherwise indicated.

AIH, autoimmune hepatitis; CI, confidence interval; CVD, cardiovascular disease; HR, hazard ratio; IQR, interquartile range; PY, person-years.

a

Conditioned on matching set within family.

b

Conditioned and further adjusted for age, sex, education and baseline medical comorbidities (CVD, non-liver cancer, diabetes, [end stage renal disease](https://www.sciencedirect.com/topics/medicine-and-dentistry/end-stage-renal-disease), and other autoimmune disease) and factors that would preclude treatment with first-line therapy (infection, psychosis, pregnancy, tuberculosis, lymphoma, and vertebral compression fracture).
